# NIR‐II Imaging‐Guided Photothermal Activation of a TRPV4‐Targeted Nanoplatform Delivering Cycloastragenol to Promote Microglia Reprogramming and α‐Synuclein Clearance in Parkinson's Disease

**DOI:** 10.1002/advs.202523380

**Published:** 2026-03-02

**Authors:** Hsuan Lo, Linjuan Feng, Shiying Li, Lik Hang Hugo Tse, Xuehan Wang, Xingyang Zhao, Shaoheng Ma, Xin Li, Yanjuan Gu, Wing‐Tak Wong

**Affiliations:** ^1^ Department of Applied Biology and Chemical Technology The Hong Kong Polytechnic University Hong Kong P. R. China; ^2^ The Hong Kong Polytechnic University Shenzhen Research Institute Shenzhen Guangdong P. R. China; ^3^ Department of Geriatrics Fujian Institute of Geriatrics Fujian Medical University Union Hospital Fuzhou P. R. China; ^4^ Fujian Key Laboratory of Molecular Neurology and Institute of Neuroscience Fujian Medical University Fuzhou P. R. China; ^5^ Department of Thoracic Surgery Guangdong Provincial People's Hospital (Guangdong Academy of Medical Sciences) Southern Medical University Guangzhou P. R. China; ^6^ Medical Research Institute Guangdong Provincial People's Hospital (Guangdong Academy of Medical Sciences) Southern Medical University Guangzhou P. R. China

**Keywords:** *α*‐synuclein clearance, microglial metabolic reprogramming, NIR‐II fluorescence/photoacoustic dual‐modality imaging, theranostic nanoplatform, TRPV4 activation

## Abstract

Current therapies for Parkinson's disease (PD) fail to concurrently address *α*‐synuclein (*α*‐syn) aggregation and microglia‐mediated neuroinflammation. Herein, we engineer a near‐infrared‐II (NIR‐II) phototheranostic nanoplatform, CAG/FD1080@MM‐aTRPV4, for synergistic regulation of microglial function and real‐time monitoring of PD pathology. We first encapsulated cycloastragenol (CAG), a bioactive compound derived from *Astragalus*, into liposomes. These liposomes were then fused with biomimetic microglial membrane‐loaded FD1080 photothermal imaging agent, followed by modification with a transient receptor potential vanilloid 4 (TRPV4)‐targeting antibody. In vitro studies using *α*‐syn‐treated cultured microglia and in vivo studies in an *α*‐syn‐overexpressing mouse model collectively demonstrate the efficacy of our strategy. It not only enables precise microglial delivery of CAG to reprogram metabolism but also sustains lysosomal function via photothermal activation of the TRPV4/CaMKK*β*/AMPK/mTOR pathway, ultimately enhancing phagocytosis. Importantly, the encapsulated FD1080 (for microglial tracking) and an anti‐*α*‐syn‐conjugated indocyanine green (anti‐*α*‐syn‐ICG) probe enable dual‐modality NIR‐II photoacoustic‐fluorescence imaging, allowing real‐time visualization of both microglial dynamics and *α*‐syn clearance. This work pioneers a photothermal immunomodulation strategy using a Chinese herb‐derived compound, presenting a versatile theranostic platform and novel mechanistic insights for microglia‐targeted PD therapy.

## Graphical abstract

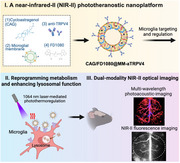

## 1 Introduction

Parkinson's disease (PD), the second most common neurodegenerative disorder, lacks disease‐modifying therapies. Its pathogenesis involves progressive accumulation of misfolded *α*‐synuclein (*α*‐syn) aggregates, whose toxic oligomers trigger mitochondrial dysfunction, synaptic damage, and endoplasmic reticulum stress while activating microglia and astrocytes to perpetuate chronic neuroinflammation [1]. Recent therapeutic advances in PD hold promise for addressing *α*‐syn pathology, with approaches such as immunotherapies and autophagy enhancers that reduce *α*‐syn aggregates and improve motor function [2, 3]. However, current treatments like L‐DOPA only alleviate symptoms without halting neurodegeneration, and conventional anti‐inflammatories (NSAIDs/corticosteroids) further pose issues of nonspecific immunosuppression [4].

While neurons are the ultimate victims of PD pathology, they possess a limited capacity to degrade extracellular *α*‐syn aggregates once the pathology spreads. In this context, microglia, as the specialized immune sentinels of the central nervous system (CNS), act as a critical double‐edged sword. They not only often fail to clear toxic aggregates in advanced PD but also can release neurotoxic cytokines. Therefore, restoring the phagocytic function of microglia to clear the pathogenic trigger “upstream,” while avoiding systemic immunosuppression, constitutes a key therapeutic strategy for modifying PD progression [5].

Microglia, the resident immune cells of the brain, dynamically survey the parenchymal microenvironment and respond to injury, infection, or homeostatic imbalance by acting as local phagocytes and damage sensors [6]. Microglial phagocytosis is a fundamental clearance pathway for neuron‐derived *α*‐syn aggregates, primarily mediated via Toll‐like receptors (TLR) and LC3‐associated endocytosis (LANDO). However, defective LC3 lipidation often impairs this process, exacerbating neuroinflammation [7, 8]. Our previous study demonstrated that cycloastragenol (CAG), a sapogenin derived from *Astragalus membranaceus* and a metabolite of astragaloside IV (AG‐IV), enhances LC3 lipidation and ameliorates fibrillar *α*‐syn‐induced autophagy impairment, signifying a promising alternative approach for PD management [9]. Additionally, microglial autophagy can be activated through multiple pathways, including rapamycin (mTOR‐dependent inducer), capsaicin targeting transient receptor potential vanilloid (TRPV) 1 receptor, and P2×7 receptor modulation [10, 11]. Among these, activation of TRPV family represents a particularly promising therapeutic strategy due to its high surface expression on microglia [12].

The TRPV4 receptor, a calcium‐permeable, mechanosensitive, and thermosensitive member of the TRP superfamily, exhibits context‐dependent activity that drives diverse physiological responses [13]. Although TRPV4 is expressed in various CNS cell types, including astrocytes, its specific role in regulating microglial motility and cytoskeletal dynamics is paramount for effective immune surveillance. Studies suggest that TRPV4 channel activity regulates motility and cytoskeletal dynamics in a temperature‐dependent manner, thereby modulating microglial activation. This mechanism critically underpins microglial motility, which enables central nervous system (CNS) surveillance and neuroprotection [14, 15, 16]. Furthermore, appropriate stimulation of the TRPV4 channel suppresses LPS‐induced abnormal microglial activation [14]. Activation of TRPV4 has been shown to increase the expression of Beclin‐1 and the LC3‐II/I ratio (key autophagy markers) as well as PINK1 and Parkin (core mitophagy proteins), thereby enhancing autophagic flux and mitophagy, thus improving mitochondrial function to facilitate recovery from ischemia/reperfusion injury [17]. In contrast, its regulation of microglial autophagy, particularly concerning *α*‐syn clearance, and the underlying mechanisms in PD pathology remain uncharacterized.

Photoacoustic microscopy (PAM) synergistically integrates optical‐resolution and acoustic‐resolution modes to achieve multiscale brain imaging [18]. By merging laser excitation with ultrasonic detection, PAM overcomes optical scattering barriers, providing cellular detail in superficial layers while enabling deep‐tissue penetration for volumetric pathophysiological mapping [19]. Unlike diffraction‐limited multiphoton microscopy or low‐resolution functional magnetic resonance imaging, PAM enables label‐free, multiparametric quantification of pathophysiological biomarkers [20]. The core principle of this technique is based on the photoacoustic effect, in which pulsed laser energy is absorbed by intrinsic chromophores such as hemoglobin or by extrinsic targeted probes [21]. This absorption induces thermoelastic expansion, resulting in the generation of ultrasonic waves. Consequently, the method allows for high‐resolution visualization of microvascular remodeling and enables the monitoring of blood oxygenation dynamics [22].

Recent advances in PAM enable comprehensive in vivo mapping of key neuroimmune structures like meningeal lymphatics and cerebral vasculature [22, 23]. Complementing this, second near‐infrared (NIR‐II, 1000–1700 nm) fluorescence imaging has emerged as a powerful modality for non‐invasive deep‐tissue diagnosis [24]. By employing high‐brightness biocompatible probes, NIR‐II imaging surmounts the limitations of conventional optical methods, including tissue absorption, autofluorescence, and photon scattering, to achieve millimeter‐scale penetration, micron‐level resolution, and a high signal‐to‐background ratio [25, 26]. While this technology has been leveraged to develop probes for diagnosing Alzheimer's disease (targeting A*β* and CTGF) [27] and Parkinson's disease (targeting copper II) in animal models [28], a NIR‐II fluorescence/photoacoustic dual‐modality imaging approach specifically targeting microglia and *α*‐syn remains unexplored.

**SCHEME 1:**
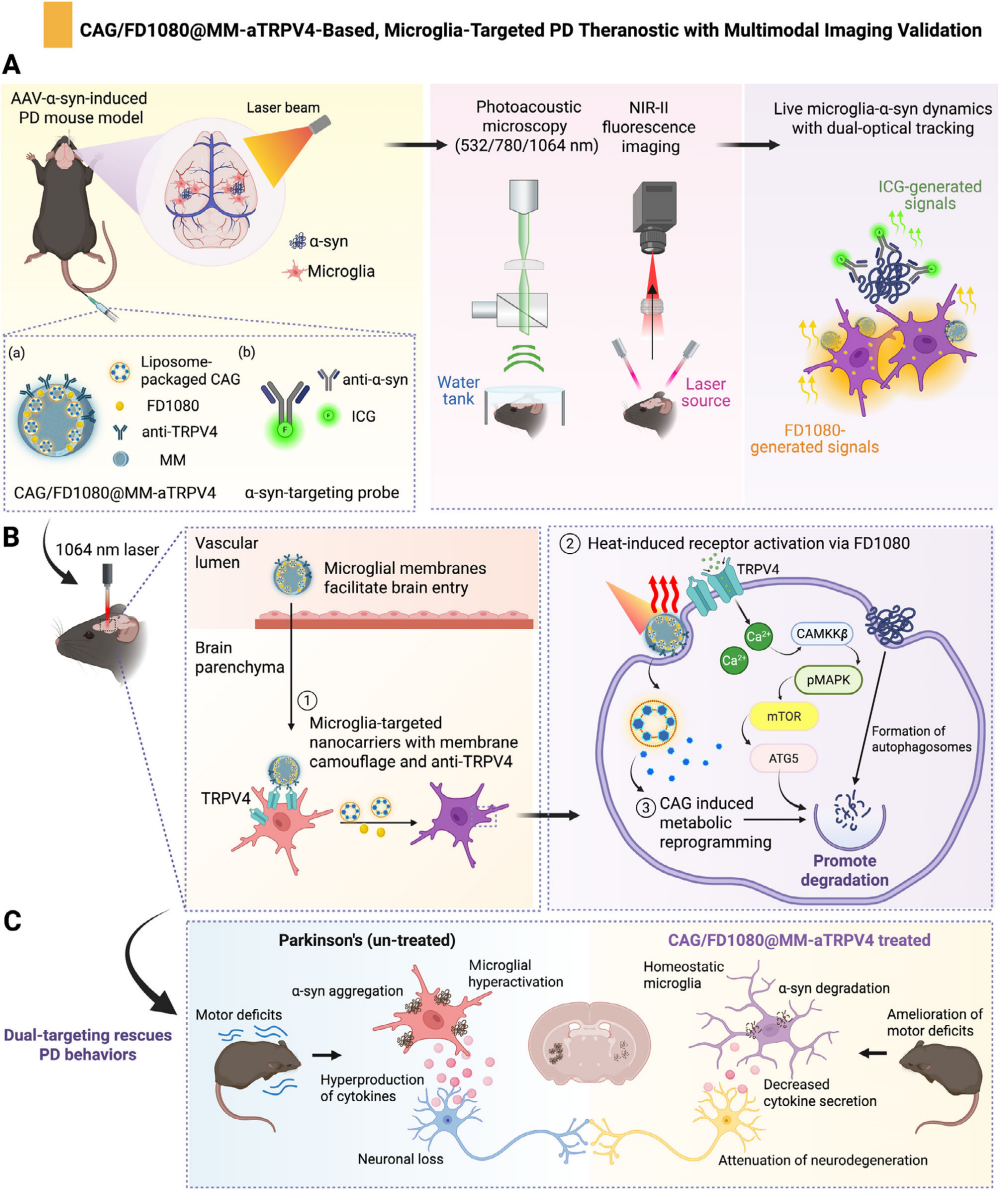
Design, brain delivery, and proposed mechanism of action of the microglia‐targeted theragnostic nanoplatform for PD therapy. (A) Schematic illustration of a theranostic system which integrates the CAG/FD1080@MM‐aTRPV4 nanoplatform (NPs, for imaging‐guided microglia regulation) with an anti‐*α*‐syn antibody‐conjugated ICG probe (for targeted *α*‐syn aggregates detection), thereby enabling dual‐modality PAM and NIR‐II fluorescence imaging to track microglia‐*α*‐syn dynamics in vivo. (B) Following systemic administration, ① the CAG/FD1080@MM‐aTRPV4 nanoplatform leverages its surface‐enriched MM and anti‐TRPV4 antibody to facilitate BBB traversal and subsequent accumulation within *α*‐syn‐affected microglia, achieved through specific binding to overexpressed TRPV4 channels. ② Subsequently, 1064 nm laser irradiation initiates therapeutic action by mediating photothermal activation of TRPV4 channels. ③ Finally, heat‐triggered TRPV4 activation synergizes with CAG‐induced metabolic reprogramming to promote microglial phagocytosis of *α*‐syn. (C) CAG/FD1080@MM‐aTRPV4 promote *α*‐syn degradation through enhanced microglial function, attenuating neuron damage and ameliorating motor deficits. This schematic was developed with BioRender.

In this study, we engineered a photothermally triggerable nanoplatform that integrates TRPV4 ion‐channel gating with CAG bioactivity to achieve spatiotemporal control of microglial function and to evaluate phagocytosis and *α*‐syn degradation in PD pathology under bidirectional regulation, using advanced imaging (Scheme 1). While acknowledging that TRPV4 is also expressed in astrocytes, our strategy specifically prioritizes microglia, the CNS's “professional phagocytes”, to enable upstream intervention against *α*‐syn toxicity. Capitalizing on the clinically established lipid nanoparticle (LNP) architecture, we engineered a platform adept at overcoming key drug delivery challenges, including solubilizing hydrophobic drugs, evading clearance, and crucially, facilitating blood‐brain barrier (BBB) transport [29, 30]. To ensure precise targeting of microglia over other TRPV4‐expressing glial cells like astrocytes, our therapeutic nanoplatform (CAG/FD1080@MM‐aTRPV4) employs a “double‐lock” targeting mechanism utilizing a biomimetic microglial membrane (MM) coating decorated with anti‐TRPV4 antibodies. This design exploits homotypic binding mechanisms to confer preferential internalization by microglia. The system co‐encapsulates the NIR‐II agent FD1080 and CAG for dual‐modality PAM/FL imaging‐guided regulation. Crucially, this multi‐modular design addresses distinct pathological bottlenecks, with MM and anti‐TRPV4 ensuring precise targeting, while FD1080 and CAG respectively initiate and sustain the clearing process. This innovative design enables precision modulation via combined photothermal‐immunotherapy, concurrently permitting deep‐tissue, dual‐wavelength photoacoustic and fluorescence visualization of microglial activation and *α*‐syn dynamics. This dual‐pronged strategy acts synergistically, whereby CAG and phototheranostic nanoregulators reprogram microglial metabolism to suppress inflammatory cytokines, while TRPV4‐mediated photothermal gating modulates the CaMKK*β*/AMPK/mTOR pathway to boost phagocytosis. Taken together, this nanoplatform pioneers the use of complementary photoacoustic and fluorescence imaging to dissect neuroimmune dynamics and integrates herbal medicine with photothermal immunomodulation, thereby offering novel synergistic therapy and insights for the treatment of PD.

## 2 Results and Discussion

### 2.1 Preparation and Characterization of an Engineered Microglia‐Targeted Biomimetic Nanoplatform

**FIGURE 1:**
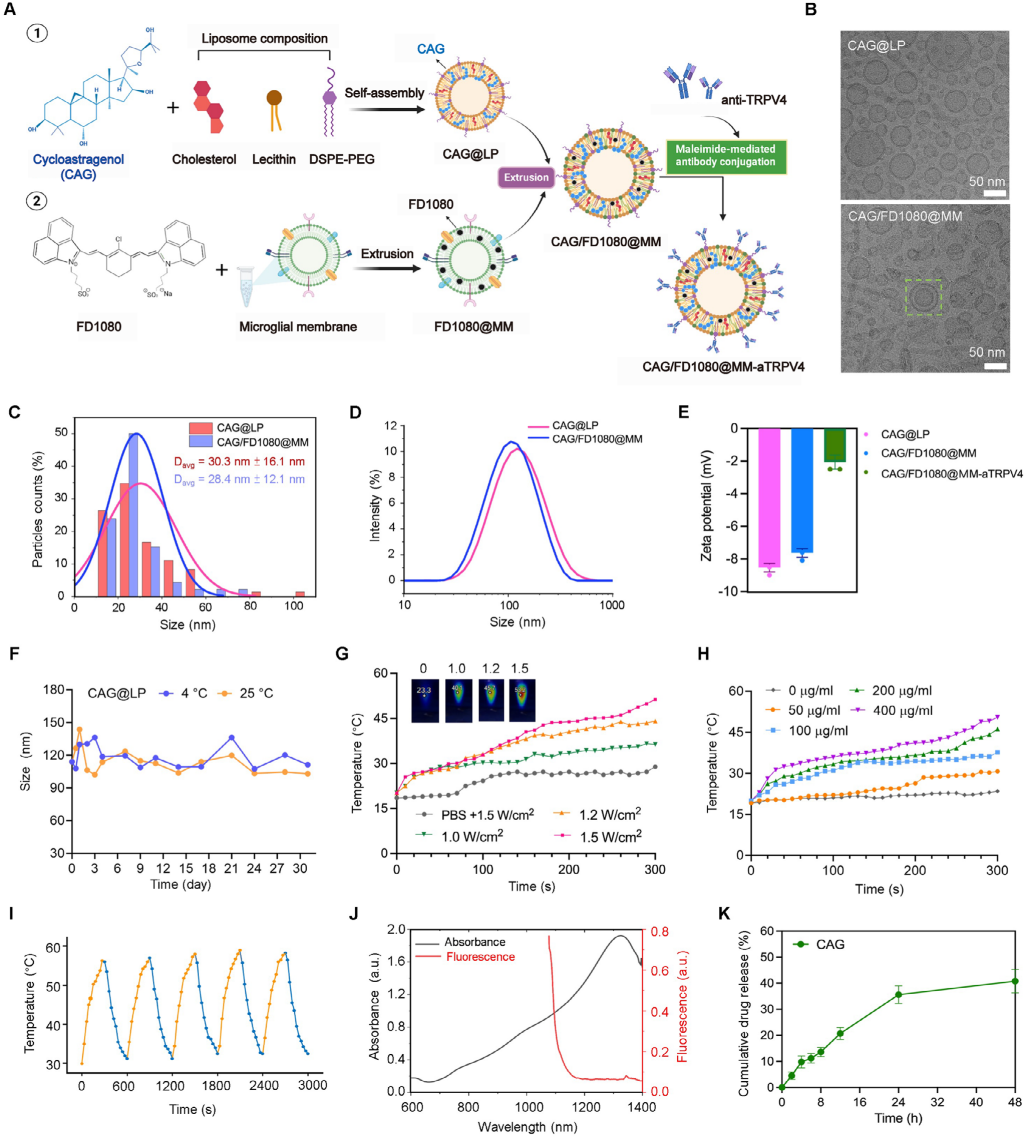
Preparation and characterization of CAG/FD1080@MM‐aTRPV4. (A) Schematic illustration of the fabrication of CAG/FD1080@MM‐aTRPV4 NPs. CAG‐encapsulated liposomes were coextruded with FD1080‐loaded MM, followed by conjugation with anti‐TRPV4 antibodies on the surface of hybrid nanovesicles for targeted delivery. (B) Cryogenic transmission electron microscopy (Cryo‐TEM) images of CAG@LP and CAG/FD1080@MM nanoplatforms. Scale bars: 50 nm. (C) Size distribution of CAG/FD1080@MM measured from Cryo‐TEM. (D) Hydrodynamic diameters of CAG@LP and CAG/FD1080@MM. (E) Zeta potential of CAG@LP, CAG/FD1080@MM and CAG/FD1080@MM‐aTRPV4. Data were expressed as means ± SD (n = 3). (F) Stability of CAG@LP size under different storage conditions. Hydrodynamic size was measured in PBS (pH 7.4) at 4°C and 25°C for 3 days using DLS. (G) Temperature rise kinetics of CAG/FD1080@MM‐aTRPV4 under NIR‐II irradiation (1064 nm) at varying power densities. (H) Concentration‐dependent photothermal effects of CAG/FD1080@MM‐aTRPV4 (1.5 W/cm^2^, 1064 nm). (I) Thermal cycling‐validated stability of CAG/FD1080@MM‐aTRPV4 (30°C ↔ 58°C). (J) UV‐vis‐NIR absorption and emission spectra of CAG/FD1080@MM‐aTRPV4 in pH 7.4 PBS buffer solution. (K) In vitro release kinetics curve of CAG@MM‐aTRPV4 in PBS (pH 7.4, 0.01 M) at 37°C with sink conditions.

The preparation of the targeted nanoplatform CAG/FD1080@MM‐aTRPV4 is summarized in Figure 1A. CAG‐encapsulated liposomes (CAG@LP) were synthesized via thin‐film hydration and subsequently fused with FD1080‐loaded MM via co‐extrusion (Figure 1A), with the MM having been extracted and purified from BV2 cells as previously described [31] (Figure S1). To ensure sufficient MM coating for effective blood‐brain barrier (BBB) translocation, the CAG/FD1080@MM nanoplatform was fabricated by co‐extruding FD1080@MM and CAG@LP at a protein‐to‐lipid mass ratio of 1:6 [32, 33]. Furthermore, an anti‐TRPV4 antibody (aTRPV4) was conjugated onto the nanoplatform surface via a sulfo‐SMCC crosslinker to enhance microglial targeting. Two control formulations, MM‐coated blank liposomes (LP@MM) and MM‐coated CAG‐loaded liposomes (CAG@MM), were established to account for nonspecific effects and isolate TRPV4‐targeted photothermal responses, respectively. This experimental design enabled a systematic evaluation to discern the individual contributions of CAG bioactivity and the active targeting mechanism.

Cryogenic transmission electron microscopy (Cryo‐TEM) analysis confirmed that both CAG@LP and CAG/FD1080@MM nanoplatforms exhibited a homogeneous spherical morphology with a core‐shell structure. Close inspection showed that CAG@LP comprised exclusively unilamellar vesicles. In contrast, the CAG/FD1080@MM nanoplatform, formed by co‐extrusion with FD1080@MM, displayed a small population of double‐bilayer vesicles (green frames). This observation indicates that most MM components were integrated into the liposomal bilayer rather than forming a superficial coating (Figure 1B). Cryo‐TEM analysis further indicated that CAG@LP and CAG/FD1080@MM had average diameters of 30.3 ± 16.1 nm and 28.4 ± 12.1 nm, respectively (Figure 1C). The slight decrease in size may be attributed to the removal of larger aggregates during the coextrusion process. Dynamic light scattering (DLS) characterization demonstrated hydrodynamic diameters of 161.6 ± 8.1 nm for CAG@LP and 150.8 ± 5.1 nm for CAG/FD1080@MM, with low polydispersity indices (PDI = 0.128 ± 0.013), indicating a uniform dispersion (Figure 1D). Zeta potential measurements yielded values of −9.22 ± 0.11 mV (CAG@LP), −8.60 ± 0.30 mV (CAG/FD1080@MM), and −2.20 ± 0.15 mV (CAG/FD1080@MM‐aTRPV4) (Figure 1E). The progressive reduction in surface charge magnitude confirmed the successful conjugation of the anti‐TRPV4 antibody to the nanoformulation. Additionally, the size of CAG@LP in PBS (pH 7.4) remained stable at 4°C (123.7 ± 11.4 nm) and 25°C (118.5 ± 16.3 nm) when monitored by DLS over 3 days (Figure 1F). The drug loading efficiency of CAG in CAG/FD1080@MM‐aTRPV4 was 11.9%, as quantified by UPLC‐QqQ‐MS.

Next, we characterized the imaging and photothermal capabilities of the CAG/FD1080@MM‐aTRPV4 nanoplatform by leveraging the superior NIR‐II photophysical properties of FD1080 [34]. Photothermal performance, a prerequisite for microglial modulation, was quantified under 1064 nm laser irradiation. Power‐dependent assays demonstrated effective photothermal saturation in CAG/FD1080@MM, reaching a ΔTmax of 53.2°C at 1.5 W/cm^2^ (Figure 1G). Concentration‐gradient studies further demonstrated a dose‐dependent photothermal response, yielding a ΔT of 0–12.7°C over 0–400 µg/mL (Figure 1H), collectively affirming its efficacy as a photothermal agent. Notably, five photothermal cycles yielded >95% retention of photothermal conversion efficiency, underscoring operational stability for therapeutic applications (Figure 1I).

Simultaneously, the CAG/FD1080@MM‐aTRPV4 nanoplatform in buffer exhibited the characteristic NIR‐II optical properties of the FD1080 fluorophore, with an absorption peak at 1300 nm and a fluorescence emission peak at 1080 nm (Figure 1J). The preservation of these spectral features confirms the aqueous stability and functional integrity of the nanoplatform, making it suitable for in vivo imaging. CAG/FD1080@MM demonstrated a sustained release profile of CAG (9.8 ± 0.7% at 4 h and 40.8 ± 2.1% at 24 h; pH 7.4, 37°C; Figure 1K), supporting its potential to meet the demand for prolonged neuroimmune modulation in PD treatment.

Finally, the nanoplatform showed excellent biocompatibility in vivo. Histology of major organs (heart, liver, spleen, lungs, kidneys, brain) showed no toxicity (Figure S2). Biochemical analysis at the maximum tolerated dose confirmed normal liver and kidney function (Figure S3), indicating its safety for systemic administration.

### 2.2 CAG@MM NPs Attenuate Microglial Activation and Promote *α*‐Syn Clearance In Vitro

**FIGURE 2:**
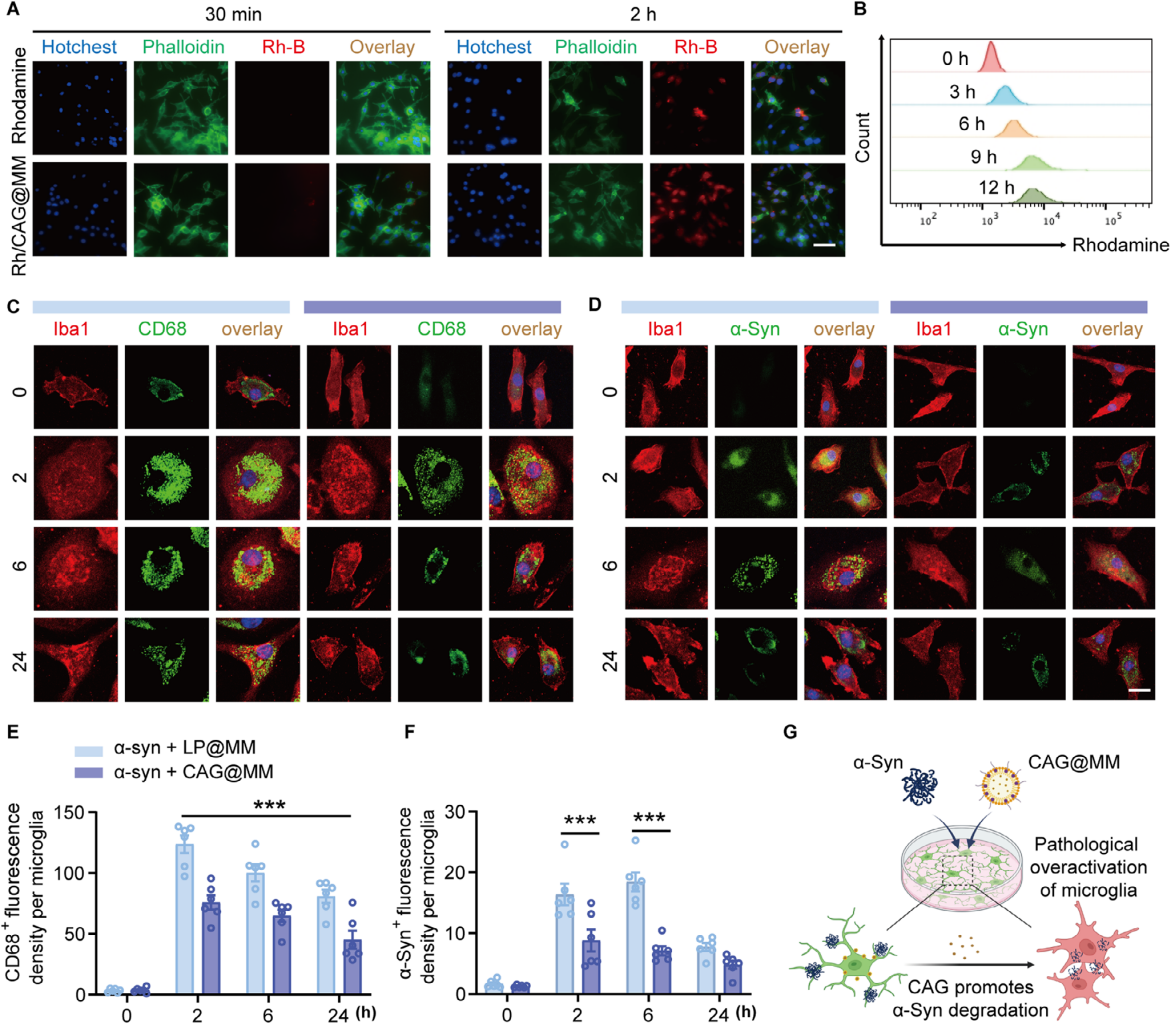
In vitro targeting validation of CAG@MM (A) Confocal microscopy images showing rhodamine B‐labeled CAG@MM (CAG/Rh@MM, red) in BV2 microglia after 30 min and 2 h incubation. Nuclei: DAPI (blue), cytoskeleton: phalloidin (green). Scale bars: 50 µm. (n = 3). (B) Flow cytometry histograms showing time‐dependent uptake (0–12 h). Rightward shift = increased internalization. Colors: 0 h (red), 3 h (blue), 6 h (orange), 9 h (green), 12 h (grey) (n = 3). (C, D) Time‐dependent changes in microglial activation and *α*‐syn degradation in *α*‐syn‐treated microglial cultures treated with LP@MM (control, empty liposome‐encapsulated MM) or CAG@MM. Confocal images of BV2 microglia at 0, 2, 6, and 24 h post‐treatment. Microglia morphology stained with Iba1 (red; specific marker for microglia), activation state labeled with anti‐CD68 (green; lysosomal marker, upregulated in activated microglia), *α*‐syn aggregation detected with anti‐*α*‐syn (green), and nuclei counterstained with DAPI (blue). Merged channels show co‐localization (yellow). (E, F) Quantification of microglial activation (E) and *α*‐syn degradation (F) based on imaging data from panels C and D. Data are shown as mean ± SEM (n = 6). (G) Schematic mechanism of CAG@MM counteracting *α*‐syn pathology. CAG@MM promotes microglia clearance of *α*‐syn and suppresses pathological overactivation of microglia. ***p < 0.001 versus *α*‐syn + LP@MM group, Two‐way ANOVA with Tukey's multiple comparisons test.

To establish the foundation for targeted microglial modulation, we first characterized the cellular internalization kinetics of our biomimetic nanoparticle platform. Confocal imaging revealed rapid BV2 microglial uptake of CAG/Rh@MM within 30 min, with prominent perinuclear accumulation by 2 h (Figure 2A). Phalloidin co‐staining confirmed active intracellular transport rather than surface adhesion, while flow cytometry quantitatively validated this time‐dependent internalization over 12 h (Figure 2B). A 3.2‐fold increase in fluorescence intensity at 12 h versus 0 h demonstrated efficient receptor‐mediated endocytosis. This process represents a critical prerequisite for achieving spatiotemporally controlled delivery.

Microglial phagocytosis is well known to promote recovery from brain injury and neurodegenerative diseases [6]. Following the establishment of efficient nanoparticle internalization, we next assessed the functional outcomes of the CAG@MM within *α*‐syn pathological contexts. To evaluate the immunomodulatory efficacy, *α*‐syn‐challenged microglia were treated with CAG@MM or control particles (LP@MM). Treatment with CAG@MM (2.45 µg/mL) produced a pronounced attenuation of pathological activation within 2–24 h, as evidenced by Iba1 staining showing reversal of somatic hypertrophy and a significant reduction in CD68 co‐localization, collectively indicating suppression of excessive activation (Figure 2C,E). Concurrently, CAG@MM significantly enhanced *α*‐syn clearance, based on two key observations from Figure 2D,F. A marked reduction in intracellular anti‐*α*‐syn immunofluorescence intensity relative to the stable fluorescence in controls was observed within 2–6 h. Furthermore, co‐localization analysis confirmed that this clearance occurred specifically following internalization (Figure 2D,F). Collectively, the evidence demonstrates that CAG@MM not only promotes *α*‐syn clearance through active phagolysosomal processing but also concurrently suppresses pathological microglial activation (Figure 2G). This suggests that CAG@MM functions through a balanced regulatory mechanism, promoting physiological phagocytosis while preventing cytotoxic overactivation, thereby restoring microglial homeostasis.

### 2.3 CAG@MM NPs Alleviates Neuroinflammation and Behavioral Deficits in *α*‐syn Overexpression Mouse Model

**FIGURE 3:**
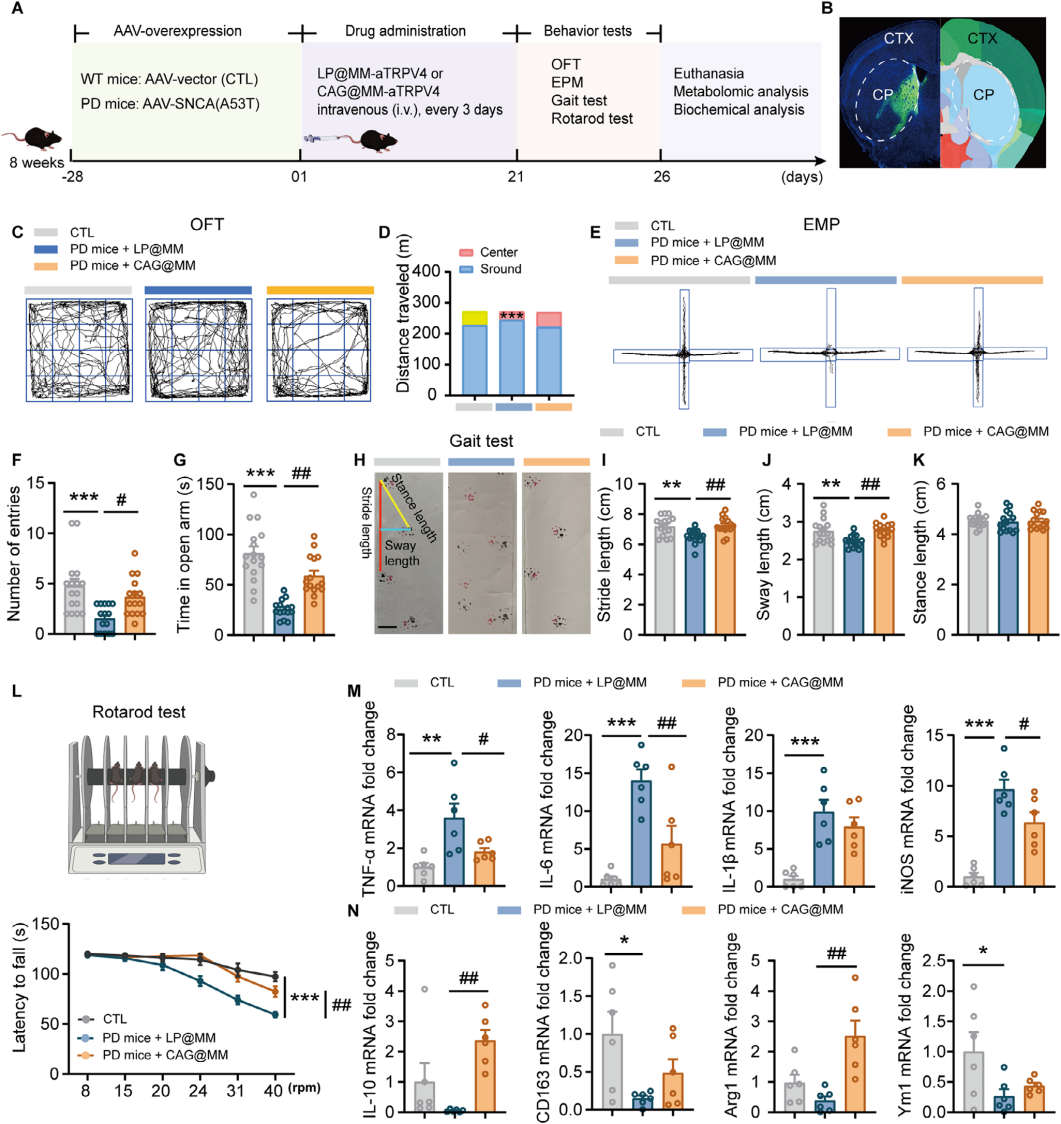
CAG@MM treatment alleviates motor deficits in PD mice. (A) Experimental timeline depicting the sequence of procedures, commencing with the injection of AAV‐vector (CTL) or AAV‐*α*‐syn (A53T) to induce PD model, followed by CAG@MM treatment, behavioral assessment, and final terminal analysis. (B) Fluorescence image showing *α*‐Syn injection sites in the striatum. (C, D) Traveled distance in the central and peripheral zones of the OFT (n = 15). (E‐G) Representative tracking traces from the EPM, with quantification of open arm entries and total time spent in open arms (n = 15). (H‐K) Schematic and statistical results of gait analysis, including stride length (I), sway length (J), and stance length (K) (n = 15). (L) Fall latency in the rotarod test (n = 15). (M‐N) mRNA expression levels of microglial pro‐inflammatory markers (TNF‐*α*, IL‐6, IL‐1*β*, iNOS) and anti‐inflammatory markers (IL‐10, CD163, Arg‐1, Ym1) across treatment groups (n = 6). Data are presented as mean ± SEM. Statistical significance was determined by one‐way ANOVA followed by Tukey's multiple comparisons test *P < 0.05, **p < 0.01, and ***p < 0.001 vs control; ^#^p < 0.05, *^##^p < 0.01* versus PD mice group.

As established evidence indicates, mutations in the SNCA gene (A53T mutation, alanine‐to‐threonine substitution at residue 53) cause familial PD by promoting the misfolding and aggregation of *α*‐syn [35]. Given the central role of *α*‐syn in pathogenesis, *α*‐syn‐based animal models gained favor in recent years for PD research [36]. To evaluate the efficacy of CAG@MM in improving motor and cognitive functions, we employed a PD mouse model using C57BL/6J mice. We induced *α*‐syn overexpression through adeno‐associated virus (AAV)‐mediated delivery of the mutant *α*‐syn A53T gene to establish the PD group, while the wild‐type control group received an empty vector. The experimental timeline is outlined in Figure 3A. Following successful model establishment, which was confirmed by striatal *α*‐syn overexpression via anti‐*α*‐syn immunofluorescence (Figure 3B), we initiated the treatment regimen. Specifically, the PD mice received systemic administration of either LP@MM or CAG@MM via tail vein injection every three days for a duration of three weeks, while the wild‐type mice were treated with saline. Behavioral assessments, including the open field (OFT) and elevated plus maze (EPM) tests, were performed 4 weeks post‐injection to evaluate motor function and anxiety‐like behavior. The results showed no significant differences in basal locomotor activity among groups, as evidenced by similar trajectory patterns in both the OFT and EPM (Figure 3C,E) and comparable total distance traveled (Figure 3D). However, PD mice treated with LP@MM exhibited pronounced anxiety‐like behavior, spending significantly less time in the OFT central zone. This phenotype was normalized by CAG@MM treatment. Consistent with these findings, LP@MM‐treated PD mice exhibited a marked anxiety‐like phenotype in the EPM, spending significantly less time in the open arms and a significant change in the number of entries (Figure 3D,G), validating heightened anxiety. In contrast, CAG@MM administration effectively mitigated these deficits, restoring behavioral performance to levels comparable to those of control mice.

We next assessed motor coordination and balance. *α*‐syn‐overexpressing mice showed significant deficits, mimicking Parkinsonian motor dysfunction [37], with altered stride length (Figure 3I), sway length (Figure 3J), and a decreased latency to fall on the rotarod (Figure 3L), but unchanged stance length (Figure 3K). CAG@MM treatment effectively rescued this motor impairment, restoring performance across all affected parameters. Together with the amelioration of anxiety‐like phenotypes, these results demonstrate that CAG@MM promotes functional recovery across motor and non‐motor domains, an effect underpinned by its enhanced targeting delivery efficiency, which facilitates the mitigation of *α*‐syn‐associated toxicity and suppression of microglia‐mediated neuroinflammation [9]. To determine whether the behavioral improvements elicited by CAG@MM were linked to attenuated neuroinflammation, we assessed microglial polarization (a key hallmark of neuroinflammation) through quantification of classic pro‐inflammatory and anti‐inflammatory markers in the striatum [38]. Treatment with CAG@MM markedly reduced the expression of pro‐inflammatory cytokines (TNF‐*α*, IL‐6, IL‐1*β*) and the pro‐inflammatory enzyme iNOS (Figure 3M). Conversely, the expression of anti‐inflammatory markers such as IL‐10 and Arg1, which were downregulated in PD mice, was restored following CAG@MM administration (Figure 3N). Taken together, these results demonstrate that CAG@MM alleviates behavioral impairments, at least in part, by rebalancing microglial polarization toward an anti‐inflammatory phenotype.

### 2.4 CAG@MM NPs Attenuate Parkinsonian Pathology by Reprogramming Striatal Metabolism

**FIGURE 4:**
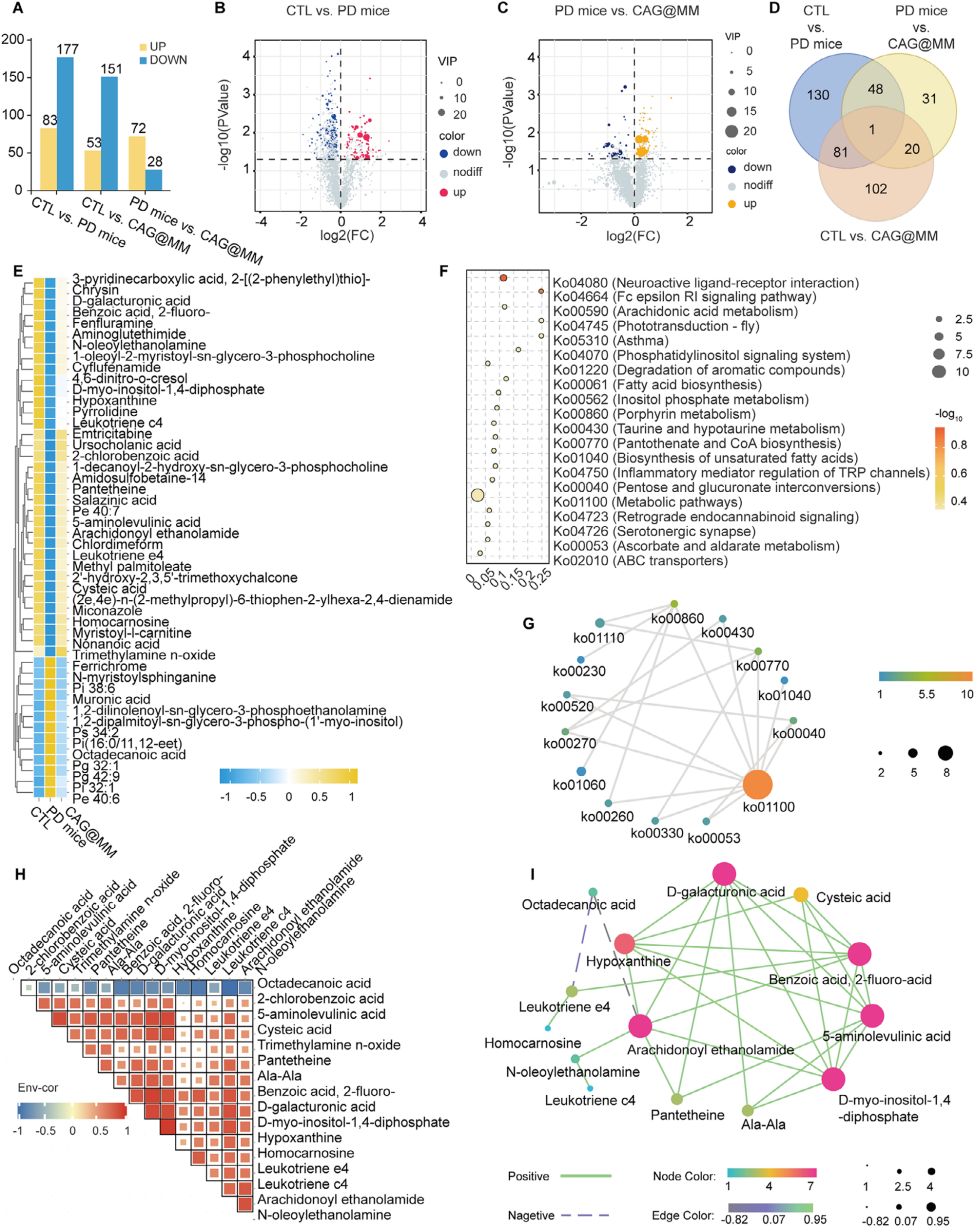
Systemic administration of CAG@MM modulates striatal metabolic reprogramming in PD mice. (A) Bar plot of significantly differential metabolites. Metabolites with significant differences between groups (p‐value < 0.05, |log_2_FC| > 1). (B) Metabolites with significant alterations (p < 0.05, |log_2_FC| > 1). Red dots represent metabolites upregulated in PD mice group (n = 83), blue dots indicate downregulated metabolites (n = 117). Thresholds marked by dashed lines. (C) Volcano plot displaying differential metabolites in Model versus CAG@MM‐treated groups. Metabolites significantly altered by treatment (p < 0.05, |log_2_FC| > 1). Orange dots denote metabolites upregulated in CAG@MM group (n = 72), purple dots show downregulated metabolites (n = 28). (D) Venn diagram quantifying shared and unique differential metabolites among CTL, PD mice, and CAG@MM‐treated groups. (E) Heatmap of expression profiles for shared differential metabolites, selected from the intersection of CTL vs. PD mice and PD mice vs. CAG@MM differential sets, across CTL, Model, and CAG@MM‐treated groups. Z‐score normalized abundance shown with hierarchical clustering. Blue to yellow gradient indicates increasing abundance. (F) KEGG pathway enrichment of intersecting differential metabolites. (G) Pathway interaction network visualized by Cytoscape showing crosstalk between KEGG pathways (FDR<0.05) from (F). (H) Metabolite Correlation Matrix. Visualization of pairwise Pearson correlations between metabolites using corrplot in R. Color gradient indicates direction: red (positive correlation), blue (negative correlation). Tile size scales with absolute correlation strength (|r|), where larger areas represent stronger correlations. (I) Metabolite‐Metabolite Interaction Network. Network constructed from pairwise Pearson correlation coefficients. Node size scales with degree centrality (connectivity). Node color gradient (blue to red) indicates the number of connected metabolites (red: high connectivity). Edge color gradient (purple to green) represents correlation strength (green: strong |r|). Edge style: Solid = positive correlation; Dashed = negative correlation (Pearson r, p < 0.05).

Emerging evidence underscores microglial metabolic reprogramming, a shift in substrate utilization between the pro‐inflammatory M1 and anti‐inflammatory M2 polarization states, as a critical driver of PD pathology [39]. Given that rectifying this metabolic imbalance can alleviate neuroinflammation and neuronal loss [40], we proposed that the protective effects of CAG@MM might be mediated through metabolic modulation. To test this hypothesis, we conducted comprehensive untargeted metabolomic profiling of striatal tissues from mice across experimental groups. Comparative metabolomic analysis revealed significant alterations in the striatal metabolic landscape across experimental groups. Specifically, multivariate analysis identified 200 dysregulated metabolites in PD mice versus controls (83 upregulated, 117 downregulated; p < 0.05, |log_2_FC| > 1; Figure 4A,B). Notably, treatment with CAG@MM NPs markedly reversed this metabolic profile, resulting in 72 upregulated and 28 downregulated metabolites compared to the PD mice (Figure 4A,C). Unsupervised clustering (PCA) of these shared differential metabolites clearly distinguished the three groups, demonstrating the robust treatment‐induced metabolic reprogramming in the striatum (Figure S4).

To pinpoint metabolites whose dysregulation was reversed by CAG@MM treatment, we performed a three‐way Venn analysis of the differential metabolite sets from the CTL vs. PD groups (PD mice), PD vs. CAG@MM groups (PD mice + CAG@MM), and CTL vs. CAG@MM groups comparisons (Figure 4D). Metabolites unique to the CTL vs. PD mice set reflect core disease‐associated disruptions. Conversely, those unique to the PD vs. CAG@MM groups set likely represent specific pharmacological effects. Importantly, the shared metabolites (48 metabolites) at the intersection of the CTL vs. PD groups and PD mice vs. CAG@MM groups comparisons represent a core set whose pathological dysregulation was effectively normalized by the treatment, highlighting key metabolic nodes critically involved in the therapeutic efficacy (Figure 4D). Consistent with this finding, a heatmap of these overlapping metabolites showed clear segregation among the CTL, PD mice, and CAG@MM‐treated groups, confirming the ability of the nanocarrier system to restore striatal metabolic homeostasis (Figure 4E). Subsequently, the heatmap of these overlapping metabolites demonstrated clear segregation among the CTL, PD mice, and CAG@MM‐treated groups, revealing the metabolites that contributed to the restoration of striatal metabolic homeostasis following nanoplatform treatment (Figure 4E).

We next sought to determine the functional implications of these metabolic shifts through KEGG pathway enrichment analysis. This analysis identified significant enrichment in pathways including neuroactive ligand‐receptor interaction, arachidonic acid metabolism, taurine and hypotaurine metabolism, and porphyrin metabolism (Figure 4F). Pathway interaction network analysis performed with Cytoscape revealed crosstalk among the enriched pathways, with the ko01100 (metabolic pathway) emerging as the central hub, thereby underscoring a systems‐level metabolic reorganization induced by CAG@MM (Figure 4G). Furthermore, correlation matrix analysis of key metabolites associated with the metabolic pathway (ko01100) revealed a network with positive and negative significant interdependencies (Figure 4H). To characterize the topology of this network, we constructed a metabolite‐metabolite interaction network. This analysis identified several high‐degree hub metabolites, including arachidonoyl ethanolamide, D‐galacturonic acid, benzoic acid, 2‐fluoro‐beta‐alanine, 5‐aminolevulinic acid, and D‐myo‐inositol‐1,4‐diphosphate (Figure 4I). Notably, these restored hubs reveal the nanoplatform's therapeutic action. Specifically, the endocannabinoid AEA, unlike pro‐inflammatory free arachidonic acid, suppresses neuroinflammation and supports neuroprotection [41]. Furthermore, elevated levels of 5‐aminolevulinic acid (essential for heme synthesis and mitochondrial respiration) and D‐myo‐inositol‐1,4‐diphosphate (a regulator of intracellular Ca^2^^+^) point to regained mitochondrial function and calcium homeostasis [42].

Together, CAG@MM reprograms striatal metabolism by establishing a protective “metabolic soil” of anti‐inflammatory lipids and restored bioenergetics, thereby attenuating Parkinsonian pathology.

### 2.5 Multi‐Wavelength Photoacoustic Imaging of CAG/FD1080@MM‐aTRPV4 Theranostic Nanoplatform In Vivo

**FIGURE 5:**
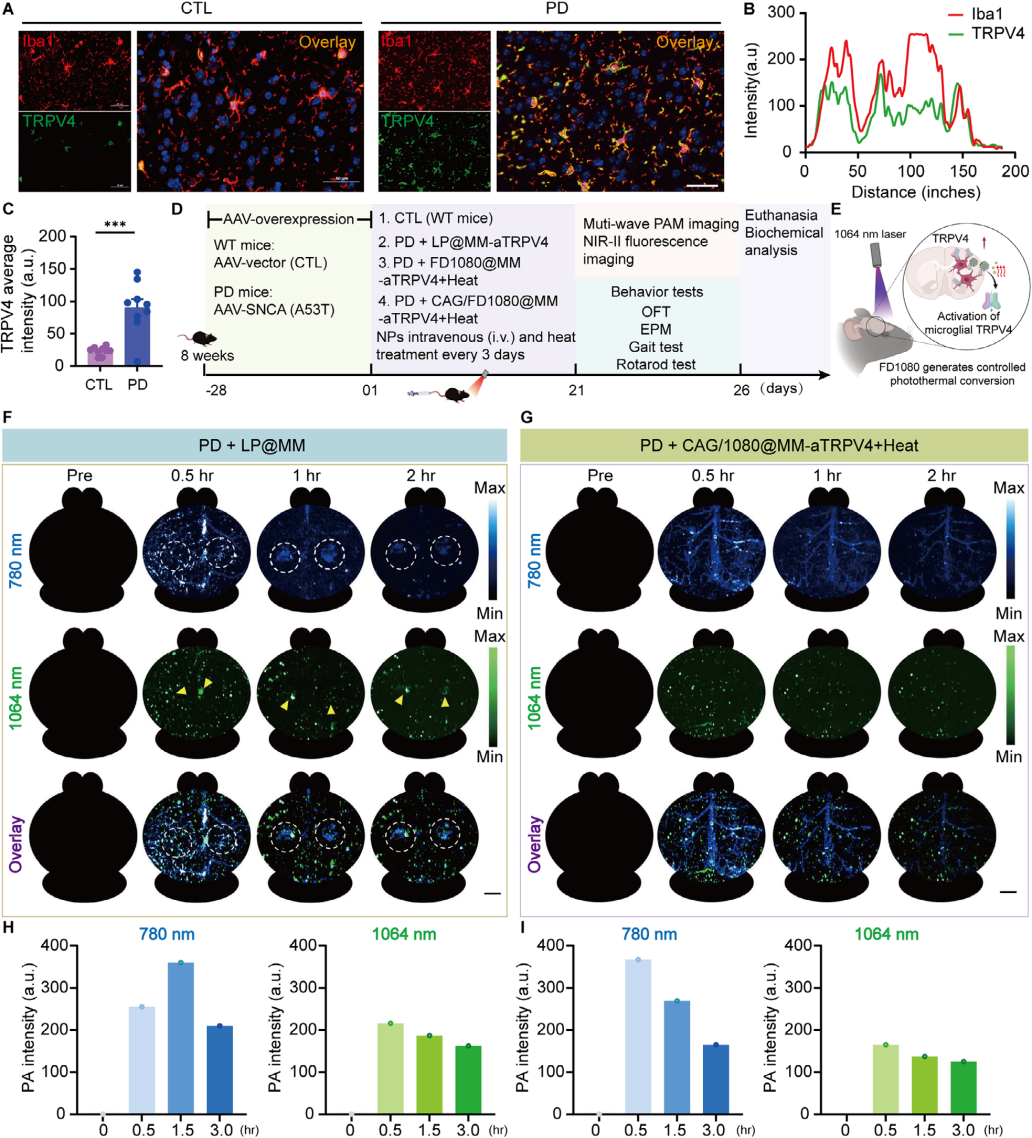
Evaluating the theranostic efficacy of CAG/FD1080@MM‐aTRPV4 nanoplatforms in PD mice via multi‐wavelength PAM. (A) Immunofluorescence analysis of TRPV4 expression (green) and Iba1^+^ microglia (red) in the substantia nigra of WT and PD mice. Yellow signal in the overlay indicates co‐localization. Scale bars: 50 µm (applicable to individual panels). (B) In PD mice, TRPV4 co‐localizes with Iba1^+^ microglia and (C) its expression is significantly upregulated. (D) Experimental design and workflow for in vivo evaluation of nanoplatforms. Mice were divided into four groups: (1) CTL (WT mice); (2) PD mice + LP@MM‐aTRPV4 (non‐therapeutic liposome control); (3) PD mice+FD1080@MM‐aTRPV4+Heat (photothermal therapy group); (4) PD mice + CAG/FD1080@MM‐aTRPV4 + Heat (combination therapy group). Nanoplatforms were administered intravenously (i.v.) followed by 1064 nm laser irradiation at the target site every three days. Therapeutic efficacy was longitudinally assessed via multi‐wavelength PAM and NIR‐II fluorescence imaging. Behavioral tests included OFT, EPM, Gait analysis, and rotarod performance. Terminal endpoint analysis involved biochemical assessment of brain tissues post‐euthanasia. (E) Illustration of photothermal activation of microglial TRPV4 channels. FD1080‐mediated photothermal conversion in NPs under 1064 nm laser irradiation generates localized heat, triggering TRPV4 gating and inducing functional modulation of microglia. (F) Representative in vivo PA images of PD mice vs. (G) nanoplatforms‐treated PD groups. Images acquired at 780 nm (*α*‐syn signal) and 1064 nm (microglial activation) across timepoints. Dashed circles: ROIs with *α*‐syn aggregates; Yellow arrows: Partially clustered microglia. Scale bar: 500 µm. (H, I) Quantitative photoacoustic analysis of *α*‐syn (780 nm) and microglial signals (1064 nm) in PD mice and NPs‐treated groups.

As noted previously, TRPV4 channels orchestrate key microglial functions by regulating morphology, motility, and cytoskeletal dynamics, thereby influencing phagocytic activity and environmental sensing capabilities [16]. Given this functional relevance, we next sought to determine whether microglial TRPV4 is involved in the pathological progression of PD by examining its expression and cellular localization in PD mice. To this end, we performed immunofluorescence staining, which revealed that TRPV4 expression was significantly upregulated in the striatum of PD mice relative to WT controls (Figure 5A,C) and that this upregulation predominantly co‐localized with Iba1^+^ microglia, a specific microglial marker (Figure 5B). Subsequently, to evaluate the targeted theranostic efficacy of microglia‐modulating nanoplatforms facilitated by photothermal TRPV4 activation, we established the therapeutic and imaging protocol summarized in Figure 5D. Mice were assigned to one of four experimental groups, including WT controls (Group 1), PD mice treated with a non‐therapeutic carrier (LP@MM‐aTRPV4, Group 2), PD mice receiving a photothermal effect‐only nanoplatform (FD1080@MM‐*α*TRPV4) plus laser irradiation (Group 3), PD mice administered the full theranostic nanoplatform (CAG/FD1080@MM‐*α*TRPV4) plus laser irradiation (Group 4). All treated groups underwent a repeated intervention every 3 days, which consisted of an intravenous injection of the respective formulation, followed by a 30‐min circulation period and subsequent 1064 nm laser irradiation at parameters (1.5 W/cm^2^, 5 min) that were validated in preliminary studies. To elucidate the mechanism underlying this theranostic approach, we proposed a model wherein FD1080‐mediated photothermal conversion generates localized heat, thereby opening microglial TRPV4 channels to induce downstream functional modulation (Figure 5E).

We employed longitudinal multi‐wavelength PAM (780 nm/1064 nm) to monitor PD pathology and therapeutic outcomes in vivo. Specifically, the 780 nm wavelength was utilized to excite the anti‐*α*‐syn‐ICG probe for detecting *α*‐syn‐related pathological changes in the brain, while the 1064 nm wavelength, matching the absorption peak of FD1080, was employed to visualize the biodistribution of the microglia‐targeted nanoplatform. This approach enabled linking of the imaging data to the therapeutic outcomes. As shown in representative images (Figure 5F,G), liposome‐treated PD mice exhibited focal *α*‐syn aggregation (dashed circles) and clustered microglial activation (yellow arrows). In contrast, animals receiving CAG/FD1080@MM‐*α*TRPV4 combined with photothermal treatment exhibited only diffuse *α*‐syn probe perfusion and a dispersed, non‐aggregated microglial distribution, indicating a restoration to a near‐physiological state (Figure 5F,G). Quantitative photoacoustic (PA) intensity analysis revealed that CAG/FD1080@MM‐*α*TRPV4 treatment converted the sustained *α*‐syn signal (780 nm) observed in PD mice into a rapid, metabolism‐like kinetic profile. In contrast, the stability of the overall microglial signal (1064 nm) indicated that the treatment specifically reduced clustered TRPV4‐expressing microglia through spatial redistribution rather than overall depletion (Figure 5H,I). Crucially, to validate that the attenuation of the 780 nm signal represents genuine clearance of pathological aggregates rather than probe pharmacokinetics or washout, these longitudinal imaging findings were corroborated by end‐point biochemical and histological analyses from the same animal cohort (as detailed in Section 2.8, Figure 8). This superior accumulation and retention implies an active targeting mechanism beyond passive leakage. By inheriting microglial surface proteins, the nanoplatform likely leverages LFA‐1/ICAM‐1 interactions [43] and LRP1‐mediated transcytosis [44] to traverse the inflamed endothelium, facilitating deep parenchymal delivery.

Together, these results underscore the value of multi‐wavelength PAM in non‐invasively validating targeted nanotherapy. The imaging outcomes directly demonstrate that the CAG/FD1080@MM‐*α*TRPV4 nanoplatform, upon photothermal activation, significantly reduces *α*‐syn aggregation in Parkinsonian pathology and alters microglial distribution pattern, thereby providing key visual evidence for its therapeutic efficacy.

### 2.6 NIR‐II Fluorescence Imaging for Longitudinal In Vivo Monitoring of Microglial‐*α*‐Syn Dynamics

**FIGURE 6:**
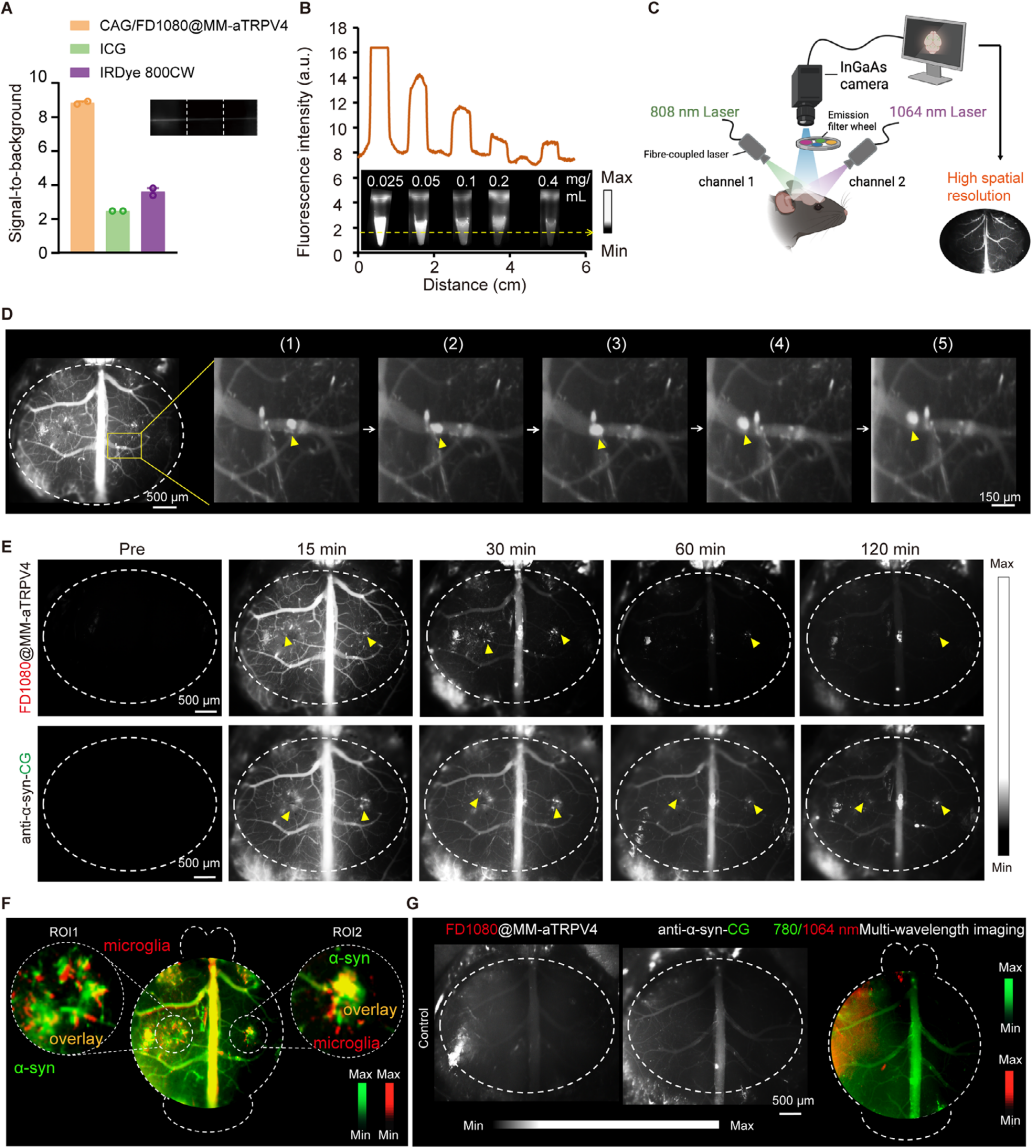
In vivo NIR‐II imaging evaluation of microglia and *α*‐Syn dynamics with high spatiotemporal resolution. (A) The histogram compares fluorescence signal‐to‐background ratios across dye and nanoplatforms groups. (B) NIR‐II fluorescence images of centrifugation tubes containing CAG/FD1080@MM‐aTRPV4 nanoplatforms at serial concentrations in FBS. Fluorescence intensity profiles (up) were acquired along the dashed lines crossing the image centers. Imaging conditions: 1064 nm excitation, 100 ms exposure, 5000 mW laser power. (C) Schematic of dual‐channel NIR‐II fluorescence imaging system. Dual‐laser excitation (808/1064 nm) with filter‐wheel emission channels (Channel 1/ Channel 2) and InGaAs camera for high‐resolution deep‐tissue NIR‐II imaging. (D) In vivo NIR‐II imaging of microglial dynamics in the mouse cortex and ROI tracking. Left: Wide‐field NIR‐II fluorescence imaging of FD1080@MM‐aTRPV4 in brain. Right: time‐series zoom‐in of the boxed ROI (images every 600 ms), showing spatiotemporal fluorescence dynamics. Scale bars: 500 µm (wide‐field image), 150 µm (ROI). (E) Longitudinal NIR‐II imaging in PD mice. Single‐frame capture showing microglial dynamics (FD1080@MM‐aTRPV4, upper) and *α*‐syn accumulation (anti‐*α*‐syn‐ICG, lower) from time‐series imaging. Scale bar: 200 µm. Imaging filter: 1000 nm LP; Exposure time: 50 ms; Excitation wavelength: 1064 and 808 nm; Imaging field of view: 4.5×4 cm^2^. (F) Reconstruction of microglia (pseudo colored red) and *α*‐syn (pseudo color green) images from (E), revealing microglia clustering around *α*‐syn deposits with colocalization appearing yellow. (G) Dual‐probe baseline NIR‐II fluorescence imaging in control mice shows FD1080@MM‐aTRPV4 targeting microglia (red, left) and anti‐*α*‐syn‐ICG targeting *α*‐syn (green, middle), with an overlay reconstruction revealing minimal colocalization (yellow, right). Scale bar: 500 µm.

To leverage its high spatiotemporal resolution [45], we employed a dual‐channel NIR‐II imaging strategy to non‐invasively track the dynamic interplay between microglia and *α*‐syn aggregates in a PD mouse model. First, to validate the imaging applicability of FD1080 beyond its photothermal function, we compared it with other fluorescent agents and nanoplatforms. Among these, the CAG/FD1080@MM‐aTRPV4 nanoplatform demonstrated superior NIR‐II imaging performance, achieving a high signal‐to‐background (SBR) ratio of ∼8 that significantly exceeded those of commercial agents such as ICG and IRDye 800CW (Figure 6A). Additionally, this nanoplatform demonstrated stable, concentration‐dependent NIR‐II fluorescence emission in fetal bovine serum (FBS), which is a medium that simulates in vivo physiological environments, across a concentration range of 0.025–0.4 mg/mL (Figure 6B). To fully leverage these favorable fluorescence properties for in vivo imaging, we established a dual‐excitation optical imaging system by integrating 808 and 1064 nm lasers with an InGaAs camera and a spectral filter wheel, thereby achieving high‐resolution, deep‐tissue imaging (Figure 6C). With this system, we performed wide‐field brain imaging to capture FD1080@MM‐*α*TRPV4‐labeled microglial dynamics. Time‐series zoom‐in imaging at 600 ms intervals further resolved fine spatiotemporal changes in fluorescence distribution (Figure 6D).

To further characterize behavior in vivo, we performed 120‐min longitudinal imaging. As shown in Figure 6E, this approach enabled us to visually distinguish microglial activation (FD1080@MM‐*α*TRPV4, upper panel) from *α*‐syn deposition (anti‐*α*‐syn‐ICG, lower panel). Both probes exhibited nearly identical accumulation dynamics, with signals peaking at 15 min and reaching optimal specificity by 30 min. Building on this synchronized temporal profile, we next assessed their spatial relationship. Overlay of the pseudo‐colored images (microglia: red; *α*‐syn: green) revealed significant colocalization (yellow regions). Acknowledging that wide‐field imaging lacks the optical sectioning to distinguish Z‐axis superposition from intracellular uptake, we interpret these findings as indicative of spatiotemporal recruitment and close proximity of FD1080‐labeled microglia to *α*‐syn aggregates (Figure 6F), rather than definitive phagocytosis. Consequently, definitive cellular‐level confirmation of engulfment was established via high‐resolution confocal microscopy and TEM, as detailed in Section 2.8 (Figure 8). In contrast, control mice showed minimal colocalization under identical imaging and dual‐probe conditions (Figure 6G). Collectively, our findings establish this NIR‐II imaging approach as a powerful method that enables real‐time visualization of microglial responses to *α*‐syn accumulation and reveals their precise spatiotemporal coordination, thereby providing new insights into neuroimmune dynamics in the living brain.

### 2.7 Thermo‐Responsive TRPV4 Nanoplatform Enables Motor Recovery in PD Mice

**FIGURE 7:**
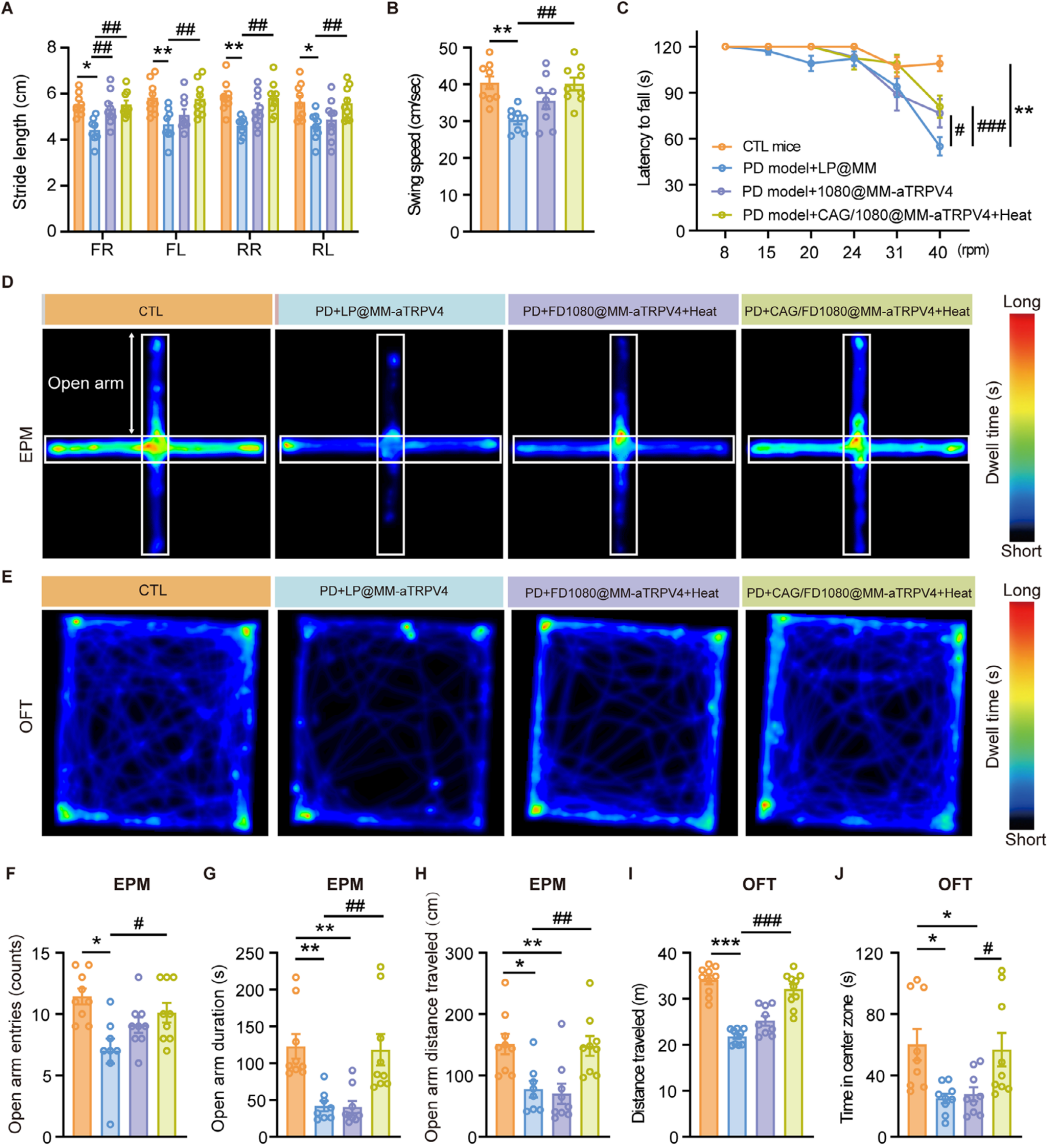
Behavioral rescue in Parkinson's mice by photothermally activated TRPV4 nanoplatforms. Post‐treatment gait analysis in mice across groups: Control (CTL), PD+LP@MM‐aTRPV4 (liposome), PD+FD1080@MM‐aTRPV4+Heat (photothermal), and PD+CAG/FD1080@MM‐aTRPV4+Heat (synergistic therapy). Photothermal activation of CAG/FD1080@MM‐aTRPV4 nanoplatforms significantly improved (A) stride length for individual paws and (B) swing speed compared to the untreated model group. Fore right (FR), Fore left (FL), Hind right (RR), and hind left (RL). (C) Improvement in motor coordination and balance was assessed by the rotarod test. (D) Anxiety and (E) exploration behaviors assessed by elevated plus maze (EPM) and open field test (OFT), with representative movement traces (upper) and locomotion heatmaps (lower). (F–H) Quantification of open arm entries (frequency), duration (s), and distance (cm). (I, J) Quantification of total distance traveled (m) and time in central zone (s) of OFT. Values are mean ± SEM (n = 8–9 mice/group). *p < 0.05, **p < 0.01 versus CTL; #p < 0.05, ^##^p < 0.01 versus PD mice; one‐way ANOVA with Tukey's post hoc test.

We next evaluated the efficacy of the CAG/FD1080@MM‐*α*TRPV4 nanoplatform combined with photothermal activation in alleviating motor and cognitive deficits in PD mice. As shown in Figure 7A,B, the treatment significantly improved gait balance parameters, including stride length of all four paws and body swing speed, compared to the untreated PD group. Furthermore, in the rotarod test assessing motor coordination, mice receiving CAG/FD1080@MM‐*α*TRPV4 plus photothermal activation exhibited a markedly prolonged latency to fall relative to PD controls (Figure 7C), indicating enhanced motor performance. Importantly, continuous monitoring throughout the therapeutic period revealed no significant body weight loss across all groups (Figure S5), ruling out systemic toxicity as a confounding factor. Beyond motor function, we assessed anxiety‐like and exploratory behaviors using the EPM and OFT. Mice treated with the combined CAG/FD1080@MM‑*α*TRPV4 photothermal regimen showed a significant reduction in anxiety‐like phenotypes, which was not observed in control groups treated with LP@MM‑*α*TRPV4 (liposome carrier) or FD1080@MM‑*α*TRPV4 with laser alone. These behavioral improvements were quantitatively supported by increased open arm entries, time spent, and distance traveled in the EPM (Figure 7F–H), as well as by elevated total distance and longer center zone duration in the OFT (Figure 7I,J).

Taken together, these behavioral results underscore the multi‐functional capability of our nanoplatform in countering PD‐related deficits. The system concurrently modulates cerebral metabolism (via CAG) and activates microglial TRPV4 (via photothermia), thereby ameliorating both motor and neuropsychiatric impairments. Although CAG/FD1080@MM‐aTRPV4 treatment significantly improved motor coordination, rotarod performance did not fully return to healthy control levels (Figure 7C). Therefore, rather than neural regeneration, the practical significance of our strategy lies in its disease‐modifying capability. By targeting the underlying pathology to arrest progression and protect surviving neurons, the nanoplatform confers substantial benefits including the robust alleviation of anxiety‐like behaviors, thereby offering a distinct translational advantage over purely symptomatic treatments. Ideally, a comprehensive comparison with representative PD therapeutic strategies further highlights the competitive advantages of our nanoplatform in terms of targeting specificity, multifunctionality, and theranostic capability (Table S3).

### 2.8 Microglial Phagocytosis Pathway Enhanced by CAG/FD1080@MM‐aTRPV4

**FIGURE 8:**
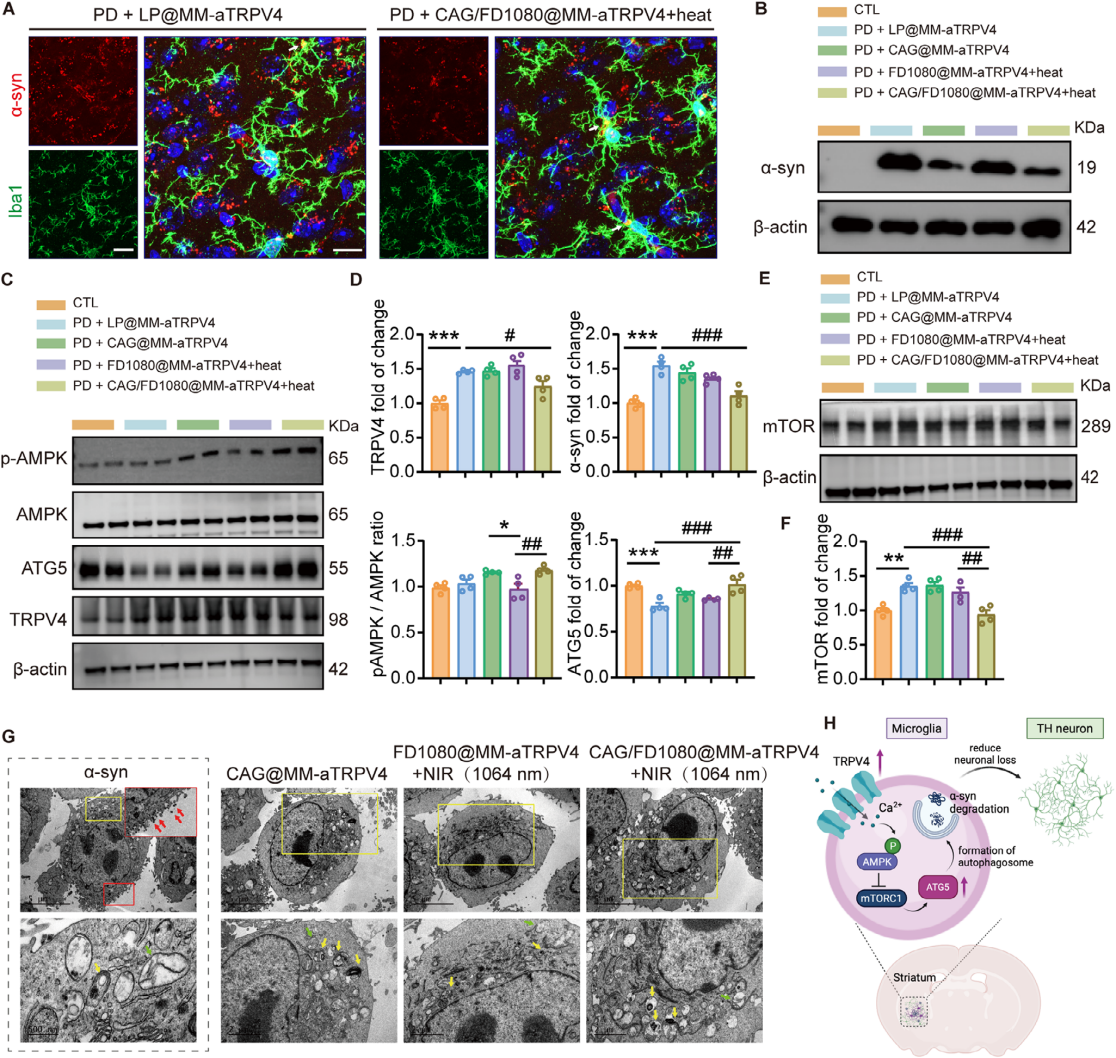
Mechanisms of microglial modulation and *α*‐syn clearance by CAG/FD1080@MM‐aTRPV4. (A) Representative immunofluorescence images of the striatum following combinatorial therapy. Top panels: Staining for pathogenic *α*‐syn aggregates (red). Bottom panels: Corresponding microglia (Iba1^+^, green) and nuclei (DAPI, blue). The PD mice treated with LP@MM‐aTRPV4 (left) exhibits abundant punctate *α*‐syn inclusions and displays microglia with a transitional morphology. In contrast, the CAG/1080@MM‐aTRPV4 + Heat group (right) exhibits a profound reduction in *α*‐syn burden, concomitant with enhanced microglial phagocytic activity, as evidenced by the apparent internalization of *α*‐syn aggregates (red) within Iba1‐positive microglia (green) (indicated by arrows). (B, C) Representative Western blot analyses of *α*‐syn, p‐AMPK, AMPK, ATG5, and TRPV4 expression in the striatum of PD mice across five groups: Control (CTL), liposome‐treated (PD+LP@MM‐aTRPV4), PD+CAG@MM‐aTRPV4, photothermal‐treated (PD+FD1080@MM‐aTRPV4+Heat), and synergistic therapy group (PD+CAG/FD1080@MM‑aTRPV4+Heat). (D) Quantitative analysis of the expression levels of the corresponding proteins. (E) mTOR protein levels. (F) Quantitative analysis of mTOR expression. (G) Representative TEM images showing microglial ultrastructure following the indicated treatments. Colored boxes indicate magnified regions of interest (Insets). Arrows highlight *α*‐syn aggregates (red), mitochondria (green), and lysosomes (yellow). Scale bars: 5 µm (overview), 1 µm (inset), and 500 nm (zoom). (H) Schematic illustration of microglia‐mediated neuroprotection via TRPV4‐Ca^2^^+^‐AMPK‐mTOR‐ATG5‐autophagy pathway against *α*‐syn toxicity.

While microglial phagocytosis is essential for brain homeostasis, its dysregulation can contribute to disease [6]. To assess functional alterations in the striatum, we performed immunofluorescence analysis, which revealed a significant enhancement in phagocytic activity following CAG/FD1080@MM‑aTRPV4 nanoplatforms treatment combined with photothermal activation in a PD mice (Figure 8A). Following treatment with LP@MM‐aTRPV4, PD mice developed widespread punctate aggregates of pathogenic *α*‐syn, correlating with microglial stress and a hyperresponsive state. Conversely, the combined CAG/FD1080@MM‑aTRPV4 and photothermal therapy facilitated *α*‐syn clearance and boosted microglial phagocytosis, accompanied by a morphological shift toward an activated phenotype. Direct evidence for this enhanced function was provided by the clear co‐localization (yellow puncta) of Iba1^+^ microglia (green) with internalized *α*‐syn (red), confirming active phagocytosis. Orthogonal fluorescence intensity profiling revealed a high degree of Iba1/*α*‐syn overlap (Figure S6), providing unambiguous evidence of intracellular engulfment rather than surface adhesion. Building on these immunohistochemical findings, Western blot analysis further confirmed that the combined CAG/FD1080@MM‑aTRPV4 and photothermal therapy significantly reduced *α*‐syn accumulation in the striatum of PD mice, compared to control groups treated with liposome or photothermal therapy alone (Figure 8B,D). This quantitative biochemical reduction directly mirrors the longitudinal decline in PA signal intensity observed in vivo (Figure 5H), thereby confirming that the non‐invasive imaging metrics serve as a reliable proxy for actual pathological clearance.

At the molecular level, this synergistic treatment markedly upregulated key autophagy‐related proteins, including the p‐AMPK/AMPK ratio, ATG5, and TRPV4 (Figure 8C,D), while suppressing mTOR expression (Figure 8E,F). To definitively establish the causal link between photothermal stimulation and this pathway activation, we verified upstream calcium dynamics using Fluo‐4 AM imaging. As shown in Figure S7, NIR irradiation of microglia treated with CAG/FD1080@MM‐aTRPV4 triggered a robust surge in intracellular calcium levels ([Ca^2^^+^]i), which was significantly higher compared to the CAG@MM‐aTRPV4 control group (Figure S7). Importantly, this calcium influx was significantly attenuated by the selective TRPV4 antagonist HC‐067047, confirming that the photothermal effect specifically gates TRPV4 channels rather than causing non‐specific membrane permeabilization. This TRPV4‐dependent calcium entry serves as the initiating signal for the established Ca^2+^/CaMKK*β*‐AMPK signaling axis. These coordinated molecular changes indicate a robust activation of the autophagic flux. Collectively, our data demonstrate that the CAG/FD1080@MM‑aTRPV4 nanoplatform, upon photothermal activation, promotes microglial phagocytosis and clearance of *α*‐syn aggregates by activating the AMPK/mTOR‐mediated autophagy pathway.

Next, we investigated whether the therapeutic efficacy was reflected in ultrastructural alterations within microglia. Initial observations in cultured microglia confirmed the presence of *α*‐syn aggregates (red arrows), which induced mitochondrial swelling and cristae disruption (Figure 8G). Comparative analysis across treatment groups demonstrated that microglia receiving the combined CAG/FD1080@MM‑aTRPV4 nanoplatform and near‐infrared irradiation (NIR; 1064 nm, 0.8 W/cm^2^) exhibited substantial improvements in subcellular architecture compared to groups treated with CAG@MM‑aTRPV4 or FD1080@MM‑aTRPV4 plus NIR alone. These ultrastructural improvements were characterized by a marked reduction in *α*‐syn aggregates (red arrows), the appearance of mitochondria with intact cristae and minimal swelling (green arrows), and a notable increase in the abundance and clarity of lysosomal compartments (yellow arrows) (Figure 8G). These prominent lysosomes further facilitate the metabolic clearance of our inherently biodegradable lipid‐based nanoplatform, ensuring safety by preventing long‐term intracellular accumulation. A critical distinction in our strategy is between specific ion channel gating and non‐specific thermal stress. Unlike general hyperthermia, which typically induces broad stress responses, our results indicate a specific TRPV4‐dependent mechanism enabled by the nanoplatform's ‘proximity effect,’ where anti‐TRPV4 conjugation ensures localized photothermal gating without high global temperatures. This specificity is biologically corroborated by the selective activation of the calcium‐dependent AMPK/mTOR axis (Figure 8C–F) and the ultrastructural restoration of mitochondria and lysosomes (Figure 8G), which fundamentally contrasts with the organelle damage typical of thermal shock.

In summary, these ultrastructural findings confirm that photothermally activated CAG/FD1080@MM‑aTRPV4 enhances microglial phagocytosis, thereby clearing pathological *α*‐syn aggregates and restoring organellar homeostasis (Figure 8H). We acknowledge that the intrastriatal AAV‐*α*‐syn model primarily captures localized synucleinopathy rather than the multi‐regional neurodegeneration of advanced PD. However, consistent with the ‘dying‐back’ hypothesis, addressing striatal dysfunction is critical for early‐stage disease modification [46]. Furthermore, by integrating TRPV4‐mediated signaling with CAG‐driven metabolic reprogramming, our dual‐mechanism strategy holds promise for broader neuroprotection beyond the striatum, warranting further validation in sporadic or multi‐hit PD models.

## 3 Conclusions

In conclusion, this study developed a novel dual‐targeting nanoplatform, CAG/FD1080@MM‑aTRPV4, which utilizes microglia membrane camouflage and anti‐TRPV4 antibody functionalization to achieve highly specific microglia‐targeted delivery and functional reprogramming. Our findings confirm that this multi‐modular architecture effectively overcomes pathological bottlenecks, with photothermal TRPV4 gating acting as the physical initiator of autophagic flux while CAG provides the sustained metabolic support required for continuous phagocytosis. By synergistically integrating CAG‐mediated bioactivity with TRPV4‐targeted photothermal activation, this system not only enhances metabolic reprogramming of microglia but also promotes phagocytic clearance of pathological *α*‐syn aggregates via modulation of the TRPV4/AMPK/mTOR/ATG5 pathway. Crucially, by empowering the “professional phagocytes” to clear toxic aggregates and mitigate neuroinflammation, this strategy confers broad neuroprotection to vulnerable neurons without requiring direct neuronal manipulation. Furthermore, preserving microglial mitochondria and enhancing lysosomal function to mitigate neuroinflammation improves motor function in PD mice. Importantly, the incorporation of NIR‐II photoacoustic and fluorescence imaging enables real‐time visualization of microglial activation and *α*‐syn clearance, offering exceptional spatiotemporal resolution for therapeutic monitoring. While the current study focuses on the intrastriatal AAV‐*α*‐syn model to rigorously validate these clearance mechanisms, future investigations will extend to sporadic PD models to further substantiate the platform's broad translational applicability. This theranostic strategy demonstrates strong translational potential by effectively merging traditional herbal medicine with advanced nanotechnology to achieve synergistic bioactivity and real‐time diagnostic feedback, showing great promise for the immunomodulatory treatment of PD and other neuroinflammatory disorders.

## 4 Experimental Section

### 4.1 Materials

Soy lecithin, 1,2‐distearoyl‐sn‐glycero‐3‐phosphoethanolamine‐N‐[methoxy (polyethylene glycol)‐2000] (DSPE‐PEG_2000_), and cholesterol were purchased from Sigma‐Aldrich. Cycloastragenol (CAG) was purchased from MACKLIN. FD1080 and ICG‐NHS ester were obtained from Xi'an Ruxi Biological Technology Co., Ltd. Sulfosuccinimidyl 4‐(N‐maleimidomethyl) cyclohexane‐1‐carboxylate (Sulfo‐SMCC) sodium and rhodamine‐NHS ester were purchased from Thermo Fisher Scientific and Merck Chemicals, respectively. Recombinant antibodies, including anti‐TRPV4 (DF8624, Affinity Biosciences) and anti‐*α*‐syn antibody (sc‐12767, Santa Cruz), were commercially sourced. All other chemicals and analytical‐grade reagents were obtained from Sigma‐Aldrich and used without further purification.

### 4.2 Synthesis of CAG/FD1080@MM

CAG‐encapsulated liposomes (CAG@LP) were first synthesized via thin‐film hydration method [47]. In brief, 31 mg of soy lecithin, 1.6 mg cholesterol, 14 mg DSPE‐PEG_2000_ and 8.75 mg of CAG was dissolved in 40 mL of chloroform. The solvent was evaporated by rotary evaporator at 30°C, forming a thin film. The thin film was rehydrated with 5 mL of PBS under bath sonication for 30 min. The unencapsulated CAG was removed by centrifugation with 4000 rpm for 10 min. The supernatant was concentrated for further application. The concentration of lipid in CAG@LP was measured by UPLC‐QqQ‐MS. Subsequently, equal volume of the FD1080@MM (7.5 mg of membrane protein) and CAG@LP at membrane protein‐to‐lipid mass ratio of 1:6 was mixed and extruded 20 cycles through polycarbonate membranes (400, 200 nm) using a mini‐extruder to yield biomimetic CAG/FD1080@MM. For the control groups, membrane‐modified liposomes (LP@MM) and membrane‐modified CAG@LP (CAG@MM) were prepared analogously.

### 4.3 Synthesis of CAG/FD1080@MM‐aTRPV4

To construct anti‐TRPV4 antibody‐modified nanoplatforms, anti‐TRPV4 antibody (100 µg/mL, 0.1 mL) was incubated with the bifunctional linker Sulfo‐SMCC (5 mg/mL, 0.1 mL) for 2 h at 4°C. Following the removal of excess linkers by ultrafiltration, the conjugate was added to CAG/FD1080@MM and stirred overnight to obtain biomimetic nanoplatform CAG/FD1080@MM‐aTRPV4. In the control, the FD1080@MM‐aTRPV4 and LP@MM‐aTRPV4 were formed using the above‐mentioned protocol.

To determine CAG loading efficiency, lyophilized CAG/FD1080@MM‐aTRPV4 was weighed and dissolved in methanol with 5 min sonication. CAG content was quantified by UPLC‐QqQ‐MS. Drug loading efficiency (LE) was calculated as: LE % = (M_t_ / M_n_) × 100%, where M_t_ is the mass of CAG loaded in the nanoplatform and M_n_ is the total mass of the nanoplatform. The CAG loading efficacy was estimated to be 11.9%.

### 4.4 PD Mouse Model Generation and Intravenous Nanotherapeutic Administration

Male C57BL/6 mice (8 weeks old) were randomized into control and PD model groups. The PD model group received bilateral intrastriatal injections of AAV2/9‐hSyn‐SNCA(A53T)‐*α*‐synuclein‐WPRE (4.5 × 10^1^ ^3^ genome particles/ml), while controls received AAV2/9‐hSyn‐empty vector‐WPRE. Viral suspensions (1 µL/side) were infused at 0.2 µL/min into the striatum at stereotaxic coordinates relative to bregma: anteroposterior (AP) +0.8 mm, mediolateral (ML) ±2.0 mm, dorsoventral (DV) −2.2 mm ventral from dura, following established protocols [9]. Four therapeutic intervention groups were established: PD model + LP@MM‐aTRPV4, PD model + CAG@MM‐aTRPV4, PD model + FD1080@MM‐aTRPV4 + Heat, and PD model + CAG/FD1080@MM‐aTRPV4 + Heat. These groups received intravenous injections of their respective nanoplatforms (5 mg/kg of CAG, 100 µL) every three days for 3 weeks, with the FD1080‐containing groups undergoing localized heat activation 45°C for 300 s during administration. Mice were maintained under controlled conditions (25°C, 55% humidity, 12 h light/dark cycle) with ad libitum access to food and water. All procedures complied with ARRIVE guidelines and were approved by The Hong Kong Polytechnic University Animal Ethics Committee (23‐24/834‐ABCT‐R‐STUDENT).

### 4.5 Untargeted Metabolomics Analysis

Snap‐frozen striatum sections (∼80 mg) were homogenized in H_2_O, extracted with methanol: acetonitrile (1:1), centrifuged (14,000 g, 4°C), vacuum‐dried, and reconstituted in acetonitrile: water (1:1) for LC‐MS/MS. Reagents included ammonium acetate (Sigma‐Aldrich), acetonitrile (Merck), ammonium hydroxide (Fisher), and methanol (Fisher). Chromatographic separation was performed on an Agilent 1290 UHPLC system coupled to a SCIEX TripleTOF 6600 Q‐TOF mass spectrometer (Shanghai Applied Protein Technology Co., Ltd.) using an ACQUITY UPLC BEH Amide column (2.1 × 100 mm, 1.7 µm; Waters) at 40°C. The mobile phase consisted of water containing 25 mM ammonium acetate/ammonium hydroxide (pH ∼10) as component A and acetonitrile as component B, delivered at 0.4 mL/min with a gradient program of 95% B (0–0.5 min), linear reduction to 65% B over 6.5 min, linear reduction to 40% B over 1 min, 1 min hold, return to 95% B over 0.1 min, and 3 min re‐equilibration. MS detection was performed using dual ESI (±) mode with ion spray voltage set at ± 5.5 kV, source temperature maintained at 600°C, and gas flow rates optimized as follows: GS1 60 psi, GS2 60 psi, CUR 30 psi. Full‐scan MS covered m/z 60–1000, while data‐dependent MS/MS scanned m/z 25–1000 using information‐dependent acquisition (IDA) with collision energy 35 ± 15 eV, declustering potential ±60 V, top 10 precursor ions per cycle, and isotope exclusion within 4 Da. Metabolite analysis was performed by Gene Denovo Biotechnology (Guangzhou) using an AB SCIEX TripleTOF 6600 system. Subsequent bioinformatic analysis employed the Omicsmart interactive platform (http://www.omicsmart.com) for real‐time data exploration and statistical visualization.

### 4.6 In Vivo Multi Wave‐Length Photoacoustic Imaging

Mouse brains were imaged with a triple‐wavelength PAM system (Inno Laser) covering 12 × 12 × 3.75 mm^3^ at 10 mm/s fast‐axis speed (Guangdong Photoacoustic Technology Co., Ltd, China). Image acquisition required ∼10 min (wavelengths: 532/780/1064 nm). Under deep anesthesia maintained at 1.8–2.0% isoflurane delivered through a stereotaxic nose cone (O_2_ 0.6 L/min; anesthesia depth confirmed by absent pedal reflex), cranial hair was removed with clippers and Nair depilatory cream. The scalp was incised along the sagittal suture, reflected laterally, and the calvaria exposed. Bilateral craniotomies (5 mm diameter) were performed over the target cortices using a stereotaxic drill with continuous saline cooling, and applied degassed ultrasound gel to the exposed cortex and secured the head in an imaging holder. Multi‐wavelength photoacoustic excitation provided distinct contrast mechanisms: 532 nm for mapping hemoglobin‐rich vasculature, 780 nm for exciting anti‐*α*‐syn‐ICG probes (0.5 mg/kg, 100 mL), and 1064 nm for detecting CAG/FD1080‐labeled nanoplatforms (0.5 mg/kg of FD1080, 100 mL). Following intravenous injection, dual‐wavelength monitoring was conducted over 2 h. The acquired data were then processed using a MATLAB‐based pipeline, which integrated the multi‐wavelength datasets and performed 3D volume denoising with a Non‐Local Means filter, followed by 2D vascular enhancement using a Hessian‐based Jerman Frangi filter.

### 4.7 In Vivo Second Near‐Infrared (NIR‐II) Fluorescence Imaging of Mouse Brain

To investigate the in vivo NIR‐II imaging performance of the developed nanoprobes, anti‐*α*‐syn‐ICG probe (0.5 mg/kg of anti‐*α*‐syn‐ICG, 100 mL) and CAG/FD1080@MM‐aTRPV4 (0.5 mg/kg of FD1080) was i.v. injected into healthy C57BL/6 mice via tail‐vein injection. The NIR‐II in vivo imaging of the mouse brain was performed using an IRNA NIR‐II fluorescence imaging system (Guangzhou IRNA nano technology Co., Ltd, China) featuring an electronically cooled InGaAs camera coupled with a 12 mm focal length prime lens (900–1700 nm anti‐reflection coating; Tamron). Uniform illumination across the field was achieved by expanding an 808/1064 nm excitation laser (Changchun New Industries Optoelectronics Tech. Co., Ltd., China) through a lens assembly connected to a collimator. Before each imaging session, the laser spot power density was calibrated to 10–20 mW/cm^2^ with exposure times between 50–500 ms. NIR‐II fluorescence signals were isolated using appropriate long‐pass filters (950, 1000, 1200, or 1400 nm; ThorLabs, USA) as required, with the imaging field covering approximately 10 cm.

### 4.8 Behavioral Analysis

Motor and non‐motor functions were assessed during the light phase (10:00–16:00) using rotarod, balance beam, gait analysis, open field, and elevated plus maze tests, as described previously [9]. For gait analysis, mice with ink‐coated paws walked a paper‐lined runway (40 cm × 5 cm), and footprints were analyzed for stride length, width, and swing speed. In rotarod testing, mice were trained at 5 rpm before undergoing six accelerating trials (8–44 rpm) with a 2 min cutoff, recording the fall latency. For the open field test, mice were placed in the arena center for 10 min, and center (inner 40%) versus periphery travel distance was quantified. In the elevated plus maze, mice faced an open arm for a 10 min free exploration, with open‐arm entry percentage analyzed by ImageEP software (v2.6).

### 4.9 Statistical Analysis

Quantitative data were analyzed using GraphPad Prism (v10.0) and are expressed as mean ± SEM. After verifying normality, differences between groups were evaluated by two‐tailed unpaired Student's t‐test, one‐way ANOVA, or two‐way ANOVA. A *p*‐value of ≤ 0.05 was considered significant. The specific statistical test used, sample size, and *p*‐value are provided for each figure.

## Conflicts of Interest

The authors declare no conflicts of interest.

## Supporting information




**Supporting File**: advs74598‐sup‐0001‐SuppMat.docx

## Data Availability

The data that support the findings of this study are available from the corresponding author upon reasonable request.
