# Supplementary material for: NIR‐II Imaging‐Guided Photothermal Activation of a TRPV4‐Targeted Nanoplatform Delivering Cycloastragenol to Promote Microglia Reprogramming and α‐Synuclein Clearance in Parkinson's Disease
Source: Adv Sci (Weinh). 2026 Mar 2;13(26):e23380. doi: 10.1002/advs.202523380 (PMC13159104; doi:10.1002/advs.202523380)
Supplement: Supplementary file 1 — Supporting File: advs74598‐sup‐0001‐SuppMat.docx [file ADVS-13-e23380-s001.docx]

**Supporting Information**

**Photothermal Activation of TRPV4-Targeted Nanoregulators Delivering Cycloastragenol Reprograms Microglia to Clear α-Synuclein in Parkinson’s Disease with NIR-II Photoacoustic-Fluorescence Imaging Monitoring**

Hsuan Lo^1,2,#^, Linjuan Feng^3,4#^, Shiying Li^1,2#^, Lik Hang Hugo Tse^1,2^, Xuehan Wang^1,2^, Xingyang Zhao^5^, Shaoheng Ma^6^, Xin Li ^1,2^, Yanjuan Gu^1,2^*, Wing-tak Wong^1,2^*

1. Department of Applied Biology and Chemical Technology, The Hong Kong Polytechnic University, Hung Hom, Hong Kong, 000, P. R. China.

2. The Hong Kong Polytechnic University Shenzhen Research Institute, Shenzhen, 518057, Guangdong, China.

3. Department of Geriatrics, Fujian Institute of Geriatrics, Fujian Medical University Union Hospital, 29 Xinquan Road, Fuzhou 350001, China

4. Fujian Key Laboratory of Molecular Neurology and Institute of Neuroscience, Fujian Medical University, 88 Jiaotong Road, Fuzhou 350001, China

5. Department of Thoracic Surgery, Guangdong Provincial People's Hospital (Guangdong Academy of Medical Sciences), Southern Medical University, Guangzhou, 510080, P. R. China

6. Medical Research Institute, Guangdong Provincial People's Hospital (Guangdong Academy of Medical Sciences), Southern Medical University, Guangzhou 510080, China.

# These authors contributed equally to this work.

*Correspondence author

**Experimental section**

**Isolation and purification of microglial membranes (MM).** Microglial membranes were isolated from the BV2 cell line (CVCL_0182) using hypotonic lysis, freeze-thaw cycling, and differential centrifugation as shown in Supplementary Figure 1. Cells were detached by gentle scraping in PBS (pH 7.4), followed by centrifugation at 1,000 × g for 5 min at 4°C, with the subsequent pellet retained. The pellet was resuspended in hypotonic lysis buffer (10 mM Tris-HCl, pH 7.4, 1 mM EDTA, and 0.1 mM phenylmethylsulfonyl fluoride (PMSF)) and supplemented with 1× protease inhibitor cocktail, then incubated on ice for 20 min to facilitate membrane destabilization. The lysate underwent five freeze-thaw cycles involving snap-freezing in liquid nitrogen for 2 min and thawing in a 37°C water bath under gentle agitation until fully dissolved. Cytosolic debris was removed by centrifugation at 3,000 × g for 10 min at 4°C, after which the membrane fragment-enriched supernatant was collected. Crude microglial membranes were isolated via ultracentrifugation of the supernatant at 100,000 × g for 1 h at 4°C using a Type 70 Ti rotor (Beckman Coulter). The resulting membrane pellet was homogenized in sterile PBS (pH 7.4) by extrusion through a 400 nm polycarbonate membrane with 20 passes. The final membrane suspension was stored at 4°C for immediate applications or at -80°C for long-term preservation.

**Instruments.** The morphology of CAG@LP and CAG/FD1080@MM was analyzed using cryogenic transmission electron microscopy (Cryo-TEM; ThermoFisher Glacios, 200 kV). Hydrodynamic diameter and zeta potential were measured by dynamic light scattering (DLS; Malvern Zetasizer Nano ZS Zen 3600) in 10 mM PBS (pH 7.4). Colloidal stability was assessed by monitoring the hydrodynamic diameter in PBS over 3 days. The concentrations of CAG and lipid in the nanoplatforms were quantified by an Agilent 1290 UHPLC system coupled with an Agilent 6460 triple quadrupole mass spectrometer. FD1080 and ICG concentrations were determined using UV-vis-NIR spectrophotometry (Shimadzu, Japan) with standard calibration curves. The NIR-II fluorescence emission (1000–1400 nm) of CAG/FD1080@MM was recorded on a fluorescence spectrophotometer (Edinburgh Instrument, Ltd, U.K.) with an 808 nm laser excitation source and a 1000 nm long-pass emission filter.

**Synthesis of FD1080@MM.** For vesicle formation, microglial membrane fragments dispersed in PBS (7.5 mg of protein per 1 mL) were sonicated for 3 minutes in an ice bath. Then they extruded 20 cycles through polycarbonate membrane (400, 200 nm) using a mini-extruder (Avanti Polar Lipids) in the presence of FD1080 dissolved in methanol (0.2 mL, 4 mg/mL) ^[1]^. Following extrusion, the suspension was transferred into ultrafiltration (MWCO 10 kDa) to remove free FD1080.

**Synthesis of rhodamine B-labeled CAG@MM.** ​​To assess cellular uptake via fluorescence microscopy, CAG/Rh@MM was synthesized following the same protocol as CAG /FD1080@MM, substituting FD1080 with rhodamine as the fluorescent dye.​

***In vitro* release profile of CAG.** The release profile of CAG from CAG/FD1080@MM-aTRPV4 was assessed using dialysis bags (MWCO 3.5 kDa; BioDee) immersed in PBS (pH 7.4) at 37 °C under continuous agitation (100 rpm). Nanoplatforms (2 mL, 1.15 mg/mL of CAG) were loaded into pre-hydrated dialysis bags. At predetermined time intervals (2, 4, 6, 8, 12, 24, and 48 h), 0.5 mL of release medium was sampled and replaced with an equal volume of fresh PBS. Cumulative release of CAG was quantified by UPLC-QqQ-MS.

**Synthesis of anti-α-syn-ICG probes.** To construct anti-α-syn-ICG for detection of α-syn aggregates, the anti-α-synuclein antibody (200 μg/mL, 0.5 mL) was incubated with indocyanine green NHS ester (ICG-NHS) (0.5 mg/mL, 0.3 mL) in PBS (pH 7.4) for 2 h under dark conditions. The reaction mixture was transferred into ultrafiltration devices (MWCO 10 kDa) to remove unreacted ICG. The concentrations of the anti-α-syn antibody and ICG in the anti-α-syn-ICG conjugate were quantified by BCA assay (Thermo Fisher Scientific) and UV-vis-NIR spectrophotometry (Shimadzu, Japan).

**Cryo-Transmission Electron Microscopy (cryo-TEM).** To visualize the size and shape of nanoplatforms, 3.4 μl of vesicles solution at a concentration of 1*10^13^ NPs/mL were deposited onto a glow-discharged (PELCO easiGlow, Ted Pella, USA) continuous carbon coated TEM grid (EMCN, China). The grids were blotted with a filter paper for 6 s after incubated for 15 s at 4℃ with 100 % relative humidity following flash-frozen in liquid nitrogen-cooled liquid ethane using a plunge-freezing system (Vitrobot Mark IV System, Thermo Fisher, USA). Subsequently, samples were imaged on Cryo-TEM with Falcon 4i direct electron detector (Glacios 200 kV, Thermo Fisher, USA).

**Cell Culture.** BV2 microglia were cultured in high-glucose DMEM supplemented with 10% fetal bovine serum (FBS) at 37°C in a humidified 5% CO₂ atmosphere. Medium was renewed every 2-3 days through subculturing. All cell culture reagents were sourced from Gibco (USA).

**Cellular uptake experiments.** BV2 microglia were seeded in confocal dishes at a density of 1×10⁵ cells per well and cultured for 24 hours. The cells were then incubated with either Rhodamine B-labeled CAG@MMs or free Rhodamine B (1 μg/mL) at 37°C for 30 minutes or 2 hours. After incubation, the cells were fixed with 4% paraformaldehyde (PFA) for 15 minutes. Cellular structures were stained with DAPI (1 μg/mL, 10 minutes) for nuclei and Alexa Fluor 488 Phalloidin (100 nM, 30 minutes) for the cytoskeleton. Following PBS washes, intracellular fluorescence was visualized using a Zeiss LSM 780 confocal microscope (Germany). Z-stack images were acquired at 0.5 μm intervals and reconstructed with ZEN software.

For quantitative uptake analysis, BV2 microglia were treated with Rhodamine B-labeled CAG@MMs for various durations (0, 3, 6, 9, and 12 hours) at 37°C. The cells were subsequently washed with PBS, detached with trypsin/EDTA, and collected by centrifugation at 300 × g for 5 minutes. Cell pellets were resuspended in flow cytometry buffer at a density of 1×10⁶ cells per sample. Cellular uptake was quantified on a BD FACSCelesta flow cytometer (USA) using a 561 nm laser for excitation and a 580/30 nm filter for emission detection, with 10,000 events recorded per sample.

**Immunocytochemistry (ICC) and fluorescence imaging**. Paraformaldehyde-fixed cells and brain sections were immunostained following established protocols ^[2]^, incubated with primary and fluorophore-conjugated secondary antibodies (details in Table S1, Supporting Information), and imaged by confocal microscopy (63× oil objective). Images were analyzed using Fiji and Imaris.

**Western blotting analysis.** Proteins were extracted from cells and striatal tissues using RIPA buffer (50 mM Tris-HCl pH 7.4, 150 mM NaCl, 1% NP-40, 0.5% sodium deoxycholate, 0.1% SDS) supplemented with protease and phosphatase inhibitors. Protein concentration was determined by a BCA assay using BSA standards. Subsequently, 20 μg of each sample was separated by SDS-PAGE on 10% gels and transferred to 0.45 μm PVDF membranes. The membranes were blocked with 5% BSA in TBST for 1 hour at 25°C and then incubated with primary antibodies overnight at 4°C, followed by HRP-conjugated secondary antibodies (1:5,000 in 5% BSA/TBST) for 1 hour at 25°C (for antibody details, see Table S1, Supporting Information). After washing three times with TBST (10 min each), signals were developed with Super ECL Plus (Servicebio) and captured using a ChemiDoc™ MP system (Bio-Rad). Band intensities were quantified with Image Lab™ software (v6.1) and normalized to β-actin.

**Transmission electron microscopy (TEM).** Brain tissue blocks (1-mm³) were immersion-fixed in 2% PFA / 2.5% glutaraldehyde in 0.1 M PB (pH 7.4) at 4°C for 24 h. Cultured cell pellets were fixed in 4% paraformaldehyde (PFA) / 2.5% glutaraldehyde in 0.1 M cacodylate (pH 7.4) at 4°C for 1 h. All samples were post-fixed in 1% OsO₄ (2 h for brain, 1 h for cells), ethanol-dehydrated, embedded in Spurr's resin, and polymerized at 60°C for 48 h. Ultrathin sections (70 nm) were double-stained with uranyl acetate and lead citrate, and imaged using a TEM operated at 80 kV.

**Real time-quantitative polymerase chain reaction (RT-qPCR).** Total RNA was extracted from primary microglia using TRIzol™ reagent (Invitrogen) followed by chloroform-isopropanol purification ^[3]^. cDNA was synthesized from 1 μg RNA with Transcriptor First Strand cDNA Synthesis Kit (Roche, Cat# 04897030001). qPCR was performed on StepOnePlus™ (Applied Biosystems) using SYBR Green Master Mix (Roche) under cycling conditions: 55°C for 2 min, 95°C for 10 min (1 cycle); 95°C for 15 s, 60°C for 1 min (40 cycles); 95°C for 15 s. All reactions included no-template controls and were run in triplicate. Relative gene expression was calculated by the 2⁻ΔΔCt method normalized to gapdh (primers in Table S2, Supporting Information).

**Histological Analysis (H&E Staining).** The harvested major organs (heart, liver, spleen, lung, kidney, and brain) were fixed in 4% paraformaldehyde, paraffin-embedded, and sectioned at 5 μm. After deparaffinization and rehydration, the sections were stained with hematoxylin and eosin (H&E) for 2 min, further gradiently dehydrated in 95% and 100% ethanol (respectively for 5 min), and finally transparentized with xylene twice (2 min per time). The sections were mounted onto coverslips with neutral gum. Histopathological morphology was observed and imaged using an optical microscope (Olympus, Japan) to evaluate potential systemic toxicity.

**Assessment of Hepatic and Renal Functions.** Blood samples were collected via retro-orbital sinus puncture into lithium-heparinized tubes and maintained on ice for 10 min. Following centrifugation at 1000 × *g* for 6 min to isolate plasma, hepatic function biomarkers (aspartate aminotransferase, AST; alanine aminotransferase, ALT) and renal function biomarkers (blood urea nitrogen, BUN; creatinine) were quantified using a Vitros 5600 automatic biochemical analyzer (Ortho Clinical Diagnostics, USA).

**Intracellular** **Ca^2+^ imaging.** BV2 microglia were seeded on fibronectin-coated glass-bottom dishes and incubated with varying concentrations of the CAG/FD1080@MM-aTRPV4 nanoplatform or TRPV4 antagonist HC-067047 (50 nM; MedChemExpress, USA) for 24 h. For calcium signaling assessment, microglia were loaded with 4 μM Fluo-4 AM (AAT Bioquest, USA) and Pluronic F-127 in Ca^2+^ Ringer’s solution for 30 min at 37 °C, followed by a 10-min de-esterification period. Time-lapse fluorescence imaging was performed using a Leica TCS SP8 confocal laser scanning microscope (Leica Microsystems, Germany). Photothermal stimulation was triggered using a 1064 nm laser (1.5 W/cm²), and intracellular Ca^2+^ dynamics were quantified by analyzing fluorescence intensity within regions of interest (ROIs) using Fiji software (NIH, USA). Calcium signal fluctuations were normalized and expressed as the ratio of fluorescence intensity (*F*) relative to the baseline (*F/F_0_*).

**Figure S1.** Schematic of microglial membrane isolation via differential centrifugation. Microglia were first harvested by cell scraping. The collected cells were centrifuged at 4 °C, 1000×g for 5 min; thereafter, the supernatant was discarded, and hypotonic lysis buffer was added, followed by incubation on ice for 20 min. Next, the mixture was subjected to a freeze-thaw cycle (repeated 5 times): transferred to liquid nitrogen for snap-freezing (2 min) and then to a 37 °C water bath with gentle shaking until dissolved. The resulting lysate was centrifuged at 4 °C, 3000×g for 10 min to collect the supernatant and pellet (cell membrane fraction). After removing impurity proteins, the cell membrane pellet was ultracentrifuged at 4 °C, 100,000×g for 1 hour. Finally, the obtained cell membrane pellet was resuspended in PBS and homogenized by extrusion to prepare microglial membranes (MM).

**
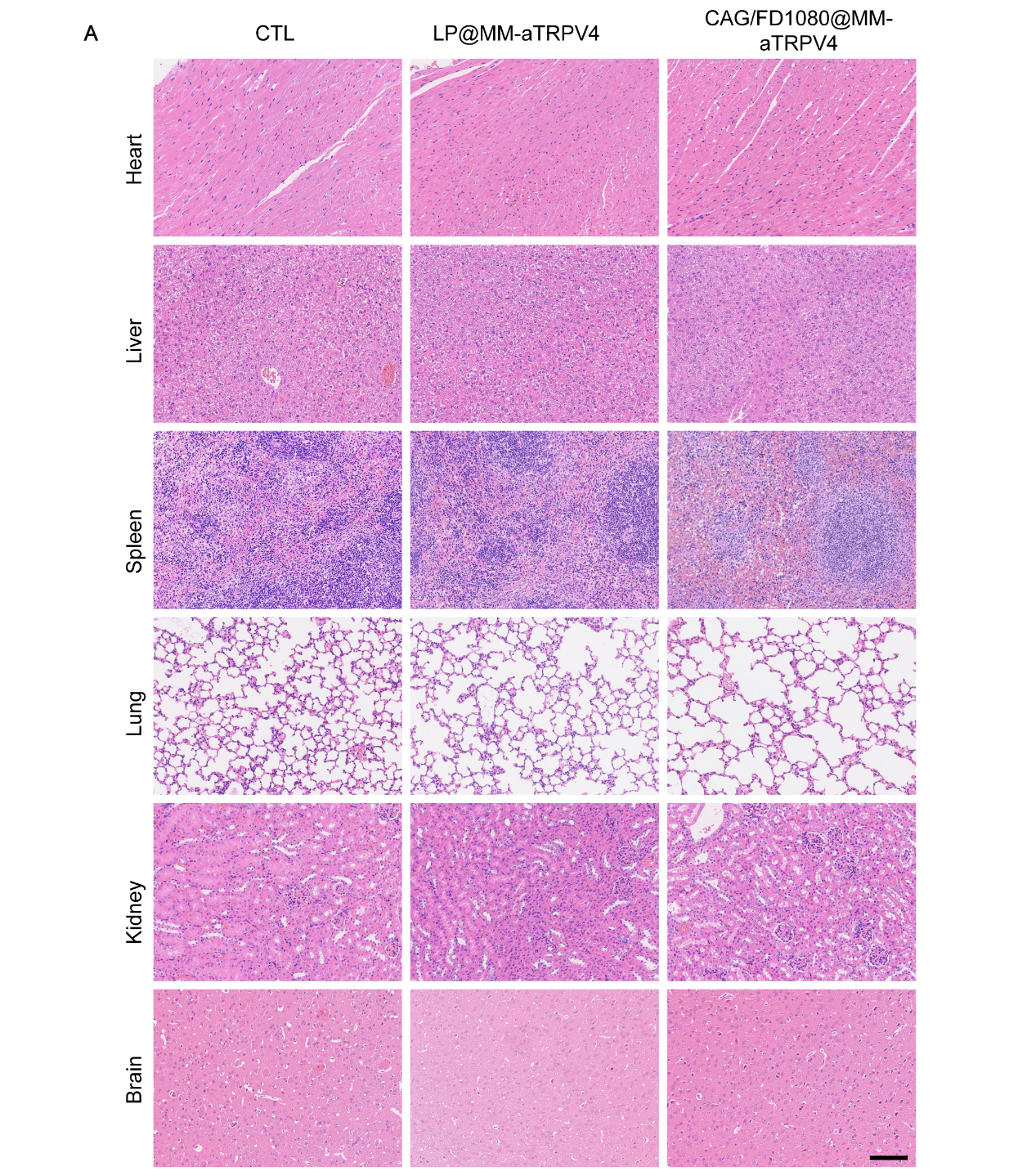
**

**Figure S2.** *In vivo* safety assessment of the nanoplatforms. (A) Representative H&E-stained sections of major organs (heart, liver, spleen, lung, kidney, and brain) harvested from mice in the CTL, LP@MM-αTRPV4, and CAG/FD1080@MM-αTRPV4 groups. Mice received treatments every two days for a 7-day period, and tissues were collected following the final administration. Scale bar: 50 μm, (n=3).


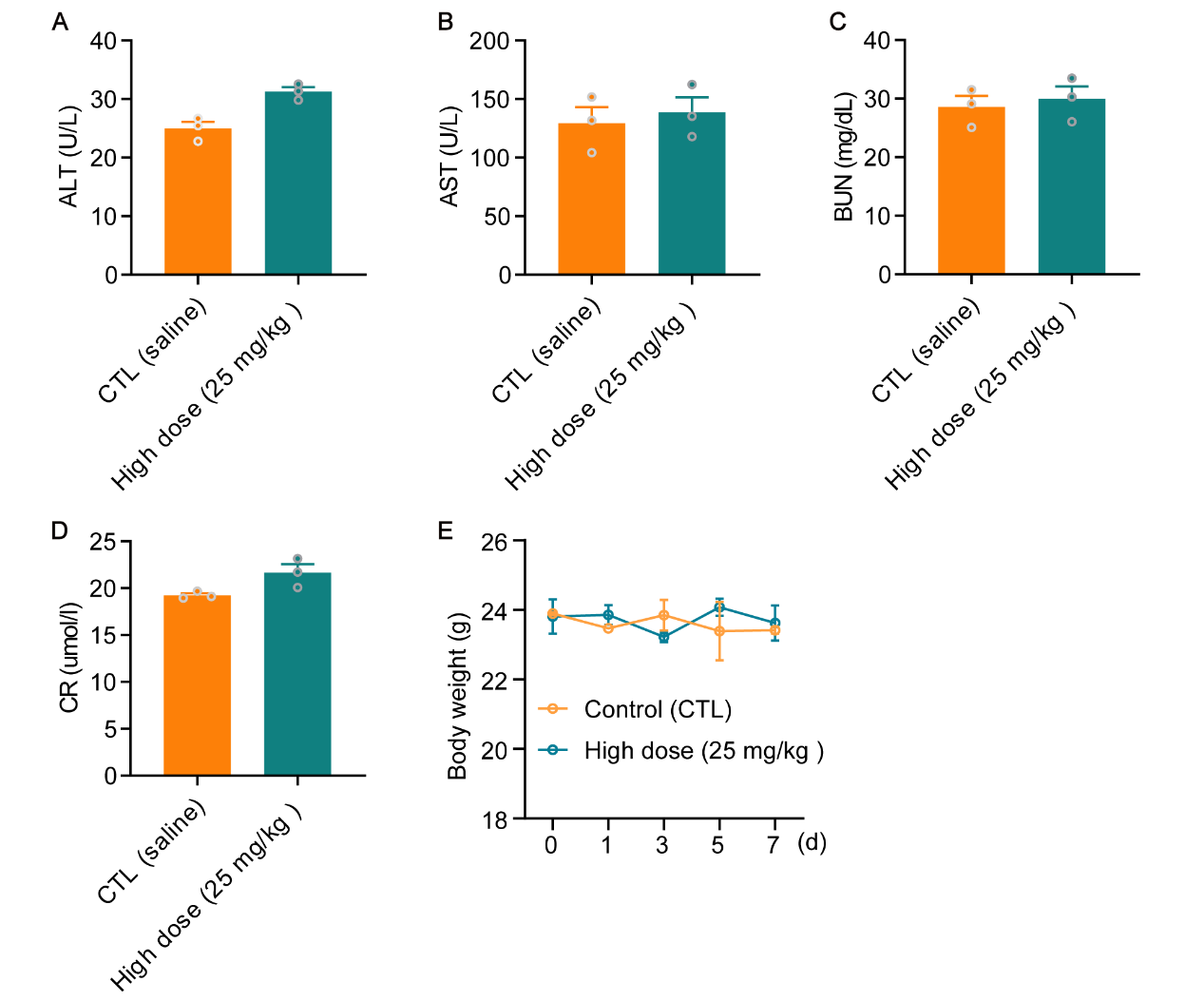


**Figure S3.** Systemic toxicity assessment at the maximum tolerated dose (MTD). C57BL/6 mice were administered a single high dose of the nanoplatform (CAG equivalent: 25 mg/kg) or saline (CTL). (A–D) Serum biochemical analysis was performed to evaluate liver and kidney function: (A) alanine aminotransferase (ALT), (B) aspartate aminotransferase (AST), (C) blood urea nitrogen (BUN), and (D) creatinine (CR). (E) Body weight monitoring over a 7-day period following high-dose administration. Data are presented as mean ± SD (n=3). Statistical significance was determined using Student's t-test (ns: not significant).

**
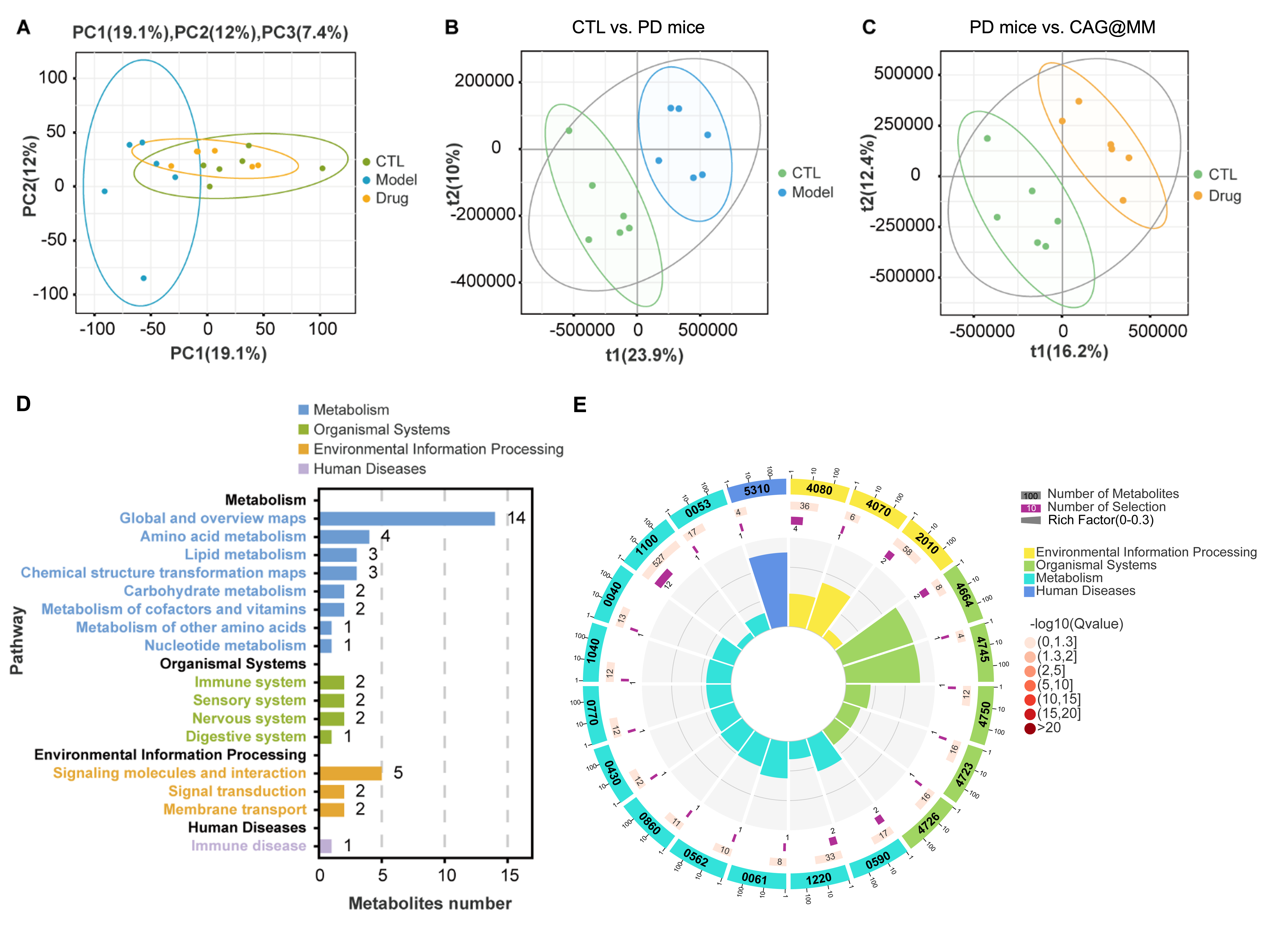
**

**Figure S4.** Metabolomic Profiling of Different Experimental Groups. (A) Principal component analysis (PCA) score plot of samples from the control (CTL), PD model (Model), and CAG@MM-treated (Drug) groups. The proportions of variance explained by PC1, PC2, and PC3 are 19.1%, 12%, and 7.4%, respectively. (B, C) Orthogonal partial least squares-discriminant analysis (OPLS-DA) score plots for the CTL vs. Model comparison (B) and the Model vs. Drug comparison (C). (D) Bar graph showing the functional classification of metabolites into major categories (Metabolism, Organismal Systems, Environmental Information Processing, Human Diseases) and their subclasses. (E) KEGG pathway annotation and enrichment analysis of differentially abundant metabolites between the Model and Drug groups. The outer ring of the chart represents the functional categories, while the inner layers display the number of metabolites, the number of selected metabolites, the rich factor (range: 0–0.3), and the -log₁₀(QValue) for each pathway, indicating the enrichment and statistical significance of altered metabolic pathways.


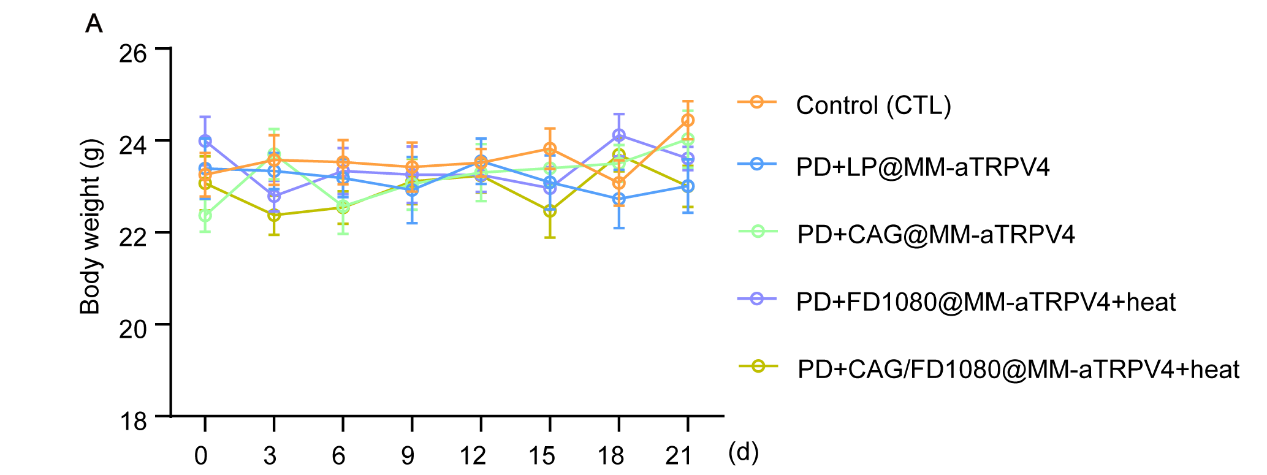


**Figure S5.** Mouse body weight by treatment group. (A) Body weight was monitored in mice over a 21-day period across the indicated treatment groups (n=8). Data are presented as mean ± SEM. Statistical analysis was performed using one-way ANOVA followed by Tukey's multiple comparisons test.

**
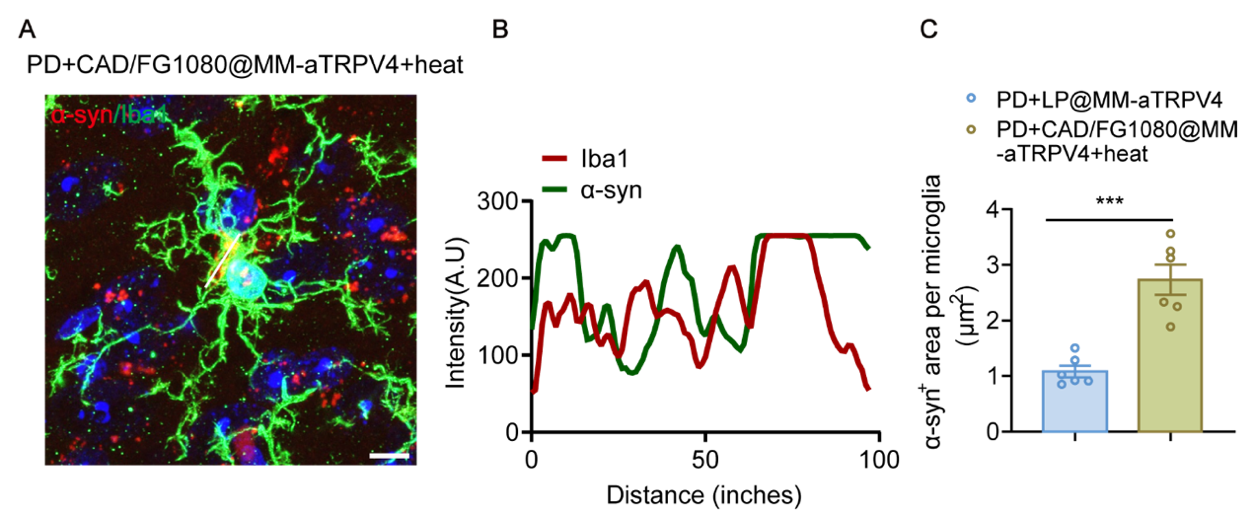
**

**Figure S6. Mechanisms of microglial modulation and** α**-syn clearance by CAG/FD1080@MM-αTRPV4. (A)** Representative immunofluorescence images of the striatum showing the colocalization of microglia (labeled with **Iba1, red**) and α*α*-syn aggregates (labeled with α**-syn, green**). Nuclei were stained with DAPI (blue). Scale bar: 10 μm. **(B)** Fluorescence intensity profile analysis along the white line drawn in (A), demonstrating the spatial correlation between Iba1 and α*α*-syn signals. **(C)** Quantification of the intracellular α-syn positive area per microglia (n = 6). Data are presented as mean ± SEM. ***p < 0.001, comparing the PD+LP@MM-αTRPV4 group with the PD+CAG/FD1080@MM-αTRPV4 + Heat group, determined by unpaired t-test.

**
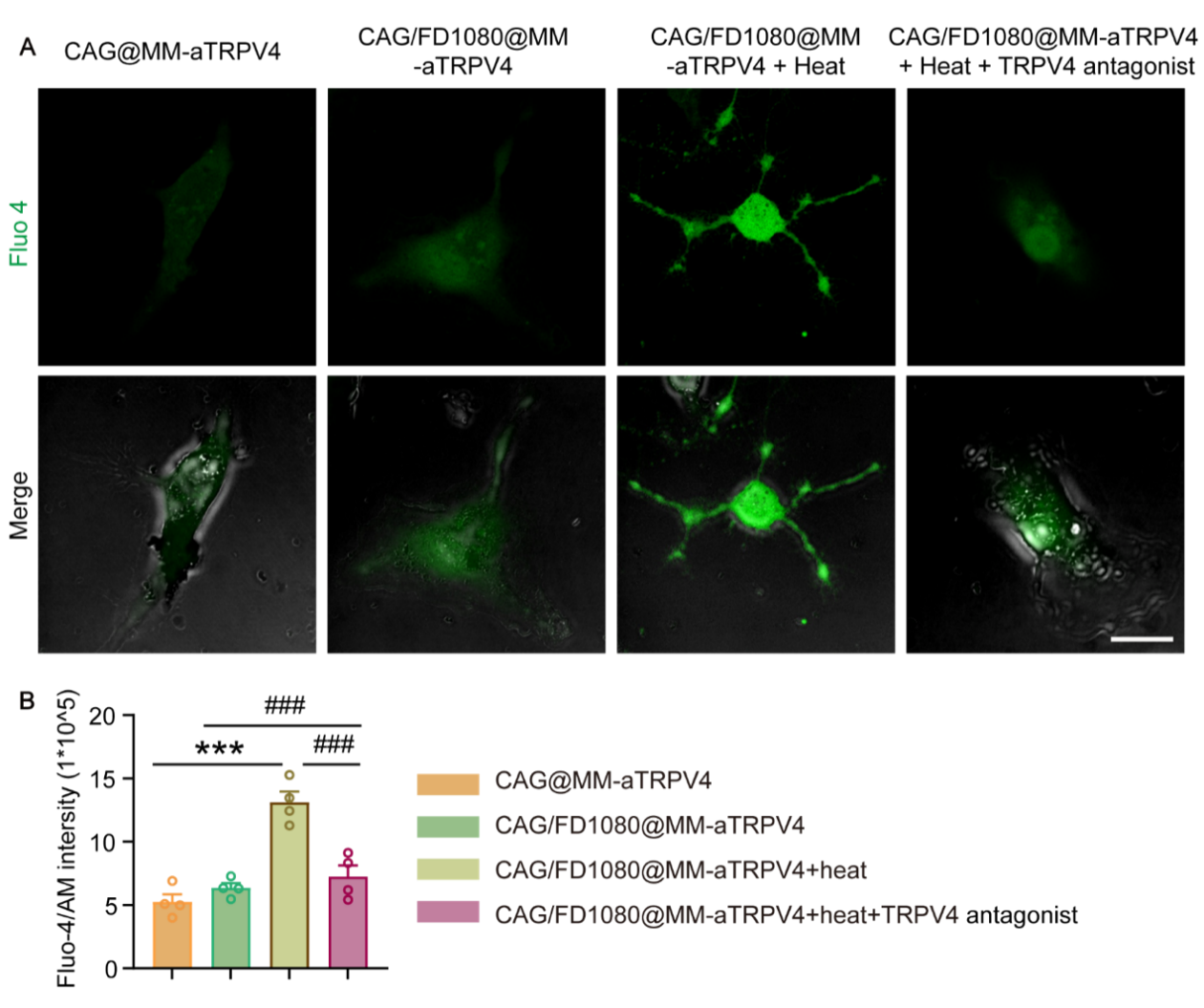
**

**Figure S7.** TRPV4 mediates the increase in intracellular Ca²⁺ levels in microglia. (A) Representative confocal fluorescence images of primary microglia loaded with the Ca²⁺ indicator Fluo-4 (green). The images display intracellular Ca²⁺ levels across the indicated treatment groups. Scale bar = 10 μm. (B) Quantification of intracellular Ca²⁺ levels based on Fluo-4 fluorescence intensity (assessed at 300 s). Data were collected from n=4 biological replicates per group (with 30–50 cells analyzed per group, 5–8 cells per well). Data are presented as mean ± SEM. ***p < 0.001 vs. CAG@MM-αTRPV4; ^###^p < 0.001 vs. CAG/FD1080@MM-αTRPV4. Statistical analysis was performed using one-way ANOVA followed by Tukey's multiple comparisons test.

**S1 Table.** The antibodies used in the IF and WB.

| **Name** | **Company** | **Cat# &** RRID | **Dilution** |
| --- | --- | --- | --- |
| **Primary antibodies** | | | |
| α-synuclein | Santa Cruz (CA, U.S.A.) | Cat# sc-12767  RRID: AB_628318 | 1:1000 |
| ATG5 | Abcam (Cambridge, UK) | Cat# AB108327  RRID: AB_2650499 | 1:1000 |
| AMPK | Cell Signal Technology  (MA, U.S.A.) | Cat#2532  RRID:AB_330331 | 1:1000 |
| β-actin | Cell Signal Technology  (MA, U.S.A.) | Cat#4967  RRID:AB_330288 | 1:5000 |
| CD68 | Bio-Rad  (MA, U.S.A.) | Cat#MCA1957  RRID:AB_322219 |  |
| GAPDH | Abcam (Cambridge, UK) | Cat# ab181602  RRID: AB_2630358 | 1:5000 |
| IBA1 | Wako Chemicals (Osaka, Japan) | Cat# 019-19741  RRID: AB_839504 | 1:500 |
| IBA1 | Abcam (Cambridge, UK) | Cat# ab5076  RRID: AB_2224402 | 1:500 |
| IBA1 | Novus (CO, U.S.A.) | Cat# NB100-1028  RRID: AB_521594 | 1:500 |
| mTOR | Santa Cruz (CA, U.S.A.) | Cat# sc-517464  RRID:AB_3186240 | 1:1000 |
| p-AMPK | Cell Signal Technology  (MA, U.S.A.) | Cat#2535  RRID: AB_331250 | 1:1000 |
| TRPV4 | Abcam (Cambridge, UK) | Cat#ab39260  RRID:AB_1143677 | 1:1000 |
| **Secondary antibodies** | | | |
| Alexa Fluor 488 (Ms) | Thermo Fisher Scientific (MA, U.S.A.) | Cat# A-21202  RRID: AB_141607 | 1:2000 |
| Alexa Fluor 488 (Rb) | Thermo Fisher Scientific (MA, U.S.A.) | Cat# A-21206  RRID: AB_2535792 | 1:2000 |
| Alexa Fluor 594 (Rb) | Thermo Fisher Scientific (MA, U.S.A.) | Cat# A-21207  RRID: AB_141637 | 1:2000 |
| Alexa Fluor 594 (Rb) | Thermo Fisher Scientific (MA, U.S.A.) | Cat# A-11012  RRID: AB_2534079 | 1:2000 |
| Alexa Fluor 594 (Ms) | Thermo Fisher Scientific (MA, U.S.A.) | Cat# A-21203  RRID: AB_141633 | 1:2000 |
| Alexa Fluor 488 (Rat) | Thermo Fisher Scientific (MA, U.S.A.) | Cat# A-21208  RRID: AB_2535794 | 1:2000 |
| Alexa Fluor 594 (Rat) | Thermo Fisher Scientific (MA, U.S.A.) | Cat# A-21209  RRID: AB_2535795 | 1:2000 |
| Alexa Fluor 594 (Goat) | Thermo Fisher Scientific (MA, U.S.A.) | Cat# A-11058  RRID: AB_142540 | 1:2000 |
| Alexa Fluor 488 (Goat) | Thermo Fisher Scientific (MA, U.S.A.) | Cat# A-32814  RRID: AB_2762838 | 1:2000 |
| Mouse HRP | Abcam (Cambridge, UK) | Cat# ab6789  RRID: AB_955439 | 1:5000 |
| Rabbit HRP | Abcam (Cambridge, UK) | Cat# ab6721  RRID: AB_955447 | 1:5000 |
| Rat HRP | Invitrogen (MA, U.S.A.) | Cat# 31470  RRID: AB_228356 | 1:5000 |
| Rat HRP | Vector Laboratories (CA, U.S.A.) | Cat# BA-9400  RRID: AB_2336202 | 1:5000 |

**S2 Table.** Forward and reverse sequences of the used primers.

| **Gene** | **GeneBank** | **Forward sequence** | **Reverse sequence** |
| --- | --- | --- | --- |
| Arg1 | [NM_001136104.1](https://www.ncbi.nlm.nih.gov/nuccore/NM_001136104.1) | AGAGCAGGATAGGATGGCAATGAC | TGGTTGAGAAGAGGTGGACACTG |
| CD163 | NM_001170395.1 | GGTGCTGGATCTCCTGGTTG | GGAGCGTTAGTGACAGCAGA |
| GAPDH | NM_008084 | CAGTGGCAAAGTGGAGATTGTTG | CTCGCTCCTGGAAGATGGTGAT |
| IL-1β | NM_008361 | ATGCCACCTTTTGACAGTGATG | TGATGTGCTGCTGCGAGATT |
| IL-6 | NM_001314054.1 | CTCCCAACAGACCTGTCTATAC | CCATTGCACAACTCTTTTCTCA |
| IL-10 | [NM_010548.2](https://www.ncbi.nlm.nih.gov/nuccore/NM_010548.2) | GGACAACATACTGCTAACCGACTC | TGGATCATTTCCGATAAGGCTTGG |
| INOS | NM_001313921.1 | ACTCAGCCAAGCCCTCACCTAC | TCCAATCTCTGCCTATCCGTCTCG |
| TNF-α | NM_001278601 | ACTGGCAGAAGAGGCACTCC | GCCACAAGCAGGAATGAGAA |
| Ym1 | NM_009892 | GCAGAAGCTCTCCAGAAGCAAT | ATTGGCCTGTCCTTAGCCCA |

**S3 Table. Comparison of CAG/FD1080@MM-aTRPV4 with representative PD therapeutic strategies.**

| **Strategy Type** | **Clinical Standards** | **Biologics (Candidate Drugs)** | **Conventional Nanomedicines** | **Our Work** |
| --- | --- | --- | --- | --- |
| **Representative Agents** | L-DOPA, Rasagiline ^[4]^ | Anti-α-syn antibodies Prasinezumab ^[5]^/  SAR446159 ^[6]^ | RVG-exosomes ^[7]^/ NK cell membrane biomimetic nanocomplex ^[8]^ | **CAG/FD1080@MM-aTRPV4** |
| **Primary Mechanism** | Dopamine replacement / MAO-B inhibition | Passive immunization (Antibody neutralization) | Anti-inflammation / Antioxidant delivery | **TRPV4-mediated Immunomodulation + Metabolic Reprogramming** |
| **Disease Modification** | **No** (Symptomatic relief only) | **Limited** (Targets only extracellular α-syn) | **Variable** (Often lacks active clearance mechanism) | **Yes** (Simultaneous α-syn clearance & inflammation resolution) |
| **BBB Penetration & Targeting** | **Variable** (Requires high doses) | Low (<0.1% brain uptake typically)  **Moderate** (next-generation, by transport) | **Moderate** (Passive or Single-ligand targeting) | **High** (Biomimetic MM + anti-TRPV4 Dual-Targeting) |
| **Imaging Capability** | None | None | Limited (Usually NIR-I or none) | **High-Resolution NIR-II PA/FL Dual-Modality** |
| **Key Limitation / Advantage** | **Limitation:** Risk of dyskinesia; does not halt progression. | **Limitation:** Poor bioavailability; single target efficacy. | **Limitation:** Lack of real-time monitoring; passive release. | **Advantage:** Light-triggered precision control; "1+1>2" synergistic efficacy. |

**Reference**

1. Li B, Lu L, Zhao M, Lei Z, Zhang F. An Efficient 1064 nm NIR-II Excitation Fluorescent Molecular Dye for Deep-Tissue High-Resolution Dynamic Bioimaging. Angew Chem Int Ed Engl. 2018; 57(25)**:** 7483-7487.

2. Feng L, Lo H, You H, Wu W, Cheng X, Xin J, Ye Z, Chen X, Pan X. Loss of cannabinoid receptor 2 promotes α-Synuclein-induced microglial synaptic pruning in nucleus accumbens by modulating the pCREB-c-Fos signaling pathway and complement system. Exp Neurol. 2023; 359**:** 114230.

3. Feng L, Lo H, Hong Z, Zheng J, Yan Y, Ye Z, Chen X, Pan X. Microglial LRRK2-mediated NFATc1 attenuates α-synuclein immunotoxicity in association with CX3CR1-induced migration and the lysosome-initiated degradation. Glia. 2023; 71(9)**:** 2266-2284.

4. Sanford M, Scott LJ. Rotigotine transdermal patch: a review of its use in the treatment of Parkinson's disease. CNS Drugs. 2011; 25(8)**:** 699-719.

5. Xiao B, Tan EK. Prasinezumab slows motor progression in Parkinsons disease: beyond the clinical data. NPJ Parkinsons Dis. 2025; 11(1)**:** 31.

6. An S, McInnis JJ, Kim D, Li Y, Tasdemir-Yilmaz O, Ahn J, Mackness BC, Kwon SH, Bonner JM, Yoo M, Dujardin S, Kim D, Park J, Yun H, Tang Y, Pradier L, Hyeon S, Song D, Sung B, Krishnan R, Spencer B, Rissman RA, Sandhu JK, Haqqani AS, Shin JW, Kim DB, Lee H, Jung J, You WK, Star AT, Delaney CE, Stanimirovic DB, Sardi SP, Lee SH, Kayatekin C. A brain-shuttled antibody targeting alpha synuclein aggregates for the treatment of synucleinopathies. NPJ Parkinsons Dis. 2025; 11(1)**:** 254.

7. Liu L, Li Y, Peng H, Liu R, Ji W, Shi Z, Shen J, Ma G, Zhang X. Targeted exosome coating gene-chem nanocomplex as "nanoscavenger" for clearing α-synuclein and immune activation of Parkinson's disease. Sci Adv. 2020; 6(50).

8. Liu J, Gao D, Hu D, Lan S, Liu Y, Zheng H, Yuan Z, Sheng Z. Delivery of Biomimetic Liposomes via Meningeal Lymphatic Vessels Route for Targeted Therapy of Parkinson's Disease. Research (Wash D C). 2023; 6**:** 0030.
